# Long Non-Coding RNA Levels Are Modulated in *Schistosoma mansoni* following *In Vivo* Praziquantel Exposure

**DOI:** 10.3390/ncrna10020027

**Published:** 2024-04-19

**Authors:** Pedro Jardim Poli, Agatha Fischer-Carvalho, Ana Carolina Tahira, John D. Chan, Sergio Verjovski-Almeida, Murilo Sena Amaral

**Affiliations:** 1 Laboratório de Ciclo Celular, Instituto Butantan, São Paulo 05503-900, SP, Brazil; pedro.poli.esib@esib.butantan.gov.br (P.J.P.); agatha.carvalho.esib@esib.butantan.gov.br (A.F.-C.); ana.tahira@butantan.gov.br (A.C.T.); verjo@iq.usp.br (S.V.-A.); 2 Global Health Institute, University of Wisconsin-Madison, Madison, WI 53792, USA; jchan32@wisc.edu; 3 Instituto de Química, Universidade de São Paulo, São Paulo 05508-900, SP, Brazil

**Keywords:** schistosomiasis, long non-coding RNAs, praziquantel, gene expression

## Abstract

Schistosomiasis is a disease caused by trematodes of the genus *Schistosoma* that affects over 200 million people worldwide. For decades, praziquantel (PZQ) has been the only available drug to treat the disease. Despite recent discoveries that identified a transient receptor ion channel as the target of PZQ, schistosome response to this drug remains incompletely understood, since effectiveness relies on other factors that may trigger a complex regulation of parasite gene expression. Long non-coding RNAs (lncRNAs) are transcripts longer than 200 nucleotides with low or no protein-coding potential that play important roles in *S. mansoni* homeostasis, reproduction, and fertility. Here, we show that *in vivo* PZQ treatment modulates lncRNA levels in *S. mansoni*. We re-analyzed public RNA-Seq data from mature and immature *S. mansoni* worms treated *in vivo* with PZQ and detected hundreds of lncRNAs differentially expressed following drug exposure, many of which are shared among mature and immature worms. Through RT-qPCR, seven out of ten selected lncRNAs were validated as differentially expressed; interestingly, we show that these lncRNAs are not adult worm stage-specific and are co-expressed with PZQ-modulated protein-coding genes. By demonstrating that parasite lncRNA expression levels alter in response to PZQ, this study unravels an important step toward elucidating the complex mechanisms of *S. mansoni* response to PZQ.

## 1. Introduction

Schistosomiasis is a neglected tropical disease caused by trematodes of the genus *Schistosoma*. Conservative estimates suggest that schistosomiasis affects over 200 million people worldwide; the disease has been reported in 78 countries [1,2,3]. Additionally, according to WHO’s Global Health Observatory data from 2020, schistosomiasis ranks among the top five leading causes of DALYs (disability-adjusted life years) and death by parasitic and vector diseases [4]. The three main species that infect humans are *Schistosoma haematobium*, *S. japonicum*, and *S. mansoni*. The latter is responsible for infection in the Americas and sub-Saharan Africa [1,5]. *S. mansoni* worms usually inhabit the mesenteric veins of their mammalian host, where males and females are paired. The females lay hundreds to thousands of fertilized eggs per day, which are carried by the circulation to the liver or pass through the intestine wall, being eliminated with the host’s feces.

Since the mid-1980s until today, treatment of schistosomiasis relies on praziquantel (PZQ), which is considered a safe and effective drug, showing only mild side effects in humans, that eliminates adult schistosome infections, often in a single dose [5,6,7,8]. For decades it has been known that PZQ affects the tegument and induces calcium influx into the whole parasite, resulting in muscular contraction and paralysis [6]. However, only very recently, the target of PZQ in *S. mansoni* has been successfully identified and characterized as a transient receptor potential melastatin ion channel, known as *Sm*.TRPM_PZQ_ [9,10]. Nevertheless, worm response to this drug is still not fully understood since it depends on a complex disruption of the parasite homeostasis. This involves the host’s immune system [1,6,7] in addition to the regulation of gene expression in the parasite when exposed to PZQ, as shown by McCusker et al. [11].

Long non-coding RNAs (lncRNAs) are transcripts longer than 200 nucleotides with low or no protein-coding potential [12,13]. In mammals, lncRNAs play a well-established role in numerous biological processes, such as gene regulation [14,15] and drug resistance [16,17]. Remarkably, lncRNAs exhibit high tissue- and species-specificity [13,14,18], hinting towards their potential as therapeutic targets against parasites [19,20]. In *S. mansoni*, the presence of lncRNAs was initially described by our group in 2011 [21] and has since been reported by other groups [22] as reviewed by Silveira et al. [19]. Furthermore, we have previously demonstrated that *S. mansoni* lncRNA levels are modulated by 5-azacytidine [23], a drug used to treat human myelodysplastic syndrome, and more recently, we highlighted their indispensable role in adult worm homeostasis and fertility [24].

Given the intricate nature of schistosomes’ response to PZQ [6,11], which remains incompletely understood, and recognizing the significant functions and regulatory roles exhibited by *S. mansoni* lncRNAs [24], we hypothesized that *S. mansoni* lncRNAs could be involved in parasite response following *in vivo* PZQ exposure. In this study, we demonstrate that *in vivo* exposure to PZQ induces significant changes in the expression of the worm’s lncRNAs, even several hours after the treatment. In our re-analyses of the RNA-Seq data of McCusker et al. [11], we found hundreds of lncRNAs differentially expressed both in mature and immature worms. Through RT-qPCR we validated the differential expression of selected lncRNAs, underscoring the impact of PZQ on altering *S. mansoni* lncRNA expression. This represents an important step toward fully elucidating the complexity of drug response in schistosomes, a crucial effort considering the emerging concern regarding PZQ-resistant worms [1,6,8].

## 2. Results

### 2.1. Sets of LncRNAs Are Differentially Expressed in S. mansoni following In Vivo Praziquantel Exposure

We re-analyzed the RNA-Seq data generated by McCusker et al. [11] to search for long non-coding RNAs (lncRNAs) differentially expressed (DE) in *S. mansoni* after *in vivo* treatment of infected mice with praziquantel (PZQ) (Supplementary Table S1 shows the samples used and alignment statistics). Three RNA-Seq datasets were re-analyzed: (i) RNA-Seq from adult worm couples obtained from mice across ten time points at 0, 0.25, 1, 3, 6, 9, 12, 24, 48, and 96 h after treatment with a single, curative dose of PZQ (400 mg/kg) delivered by oral gavage at 7 weeks post-infection (“time-course experiment”); (ii) RNA-Seq from adult worm couples obtained from mice 14 h after treatment with a single, sub-lethal dose of PZQ (100 mg/kg) delivered by intraperitoneal injection at 4 weeks post-infection (“4-weeks experiment”); (iii) RNA-Seq from adult worm couples obtained from mice 14 h after treatment with a single, sub-lethal dose of PZQ (100 mg/kg) delivered by intraperitoneal injection at 7 weeks post-infection (“7-weeks experiment”).

As expected, principal component analysis (PCA) resulted in transcriptomes of the PZQ-treated and control groups segregating broadly into two distinct regions with replicates from the same conditions clustering together, both for their control or PZQ-treated groups (Supplementary Figure S1).

**Figure 1:**
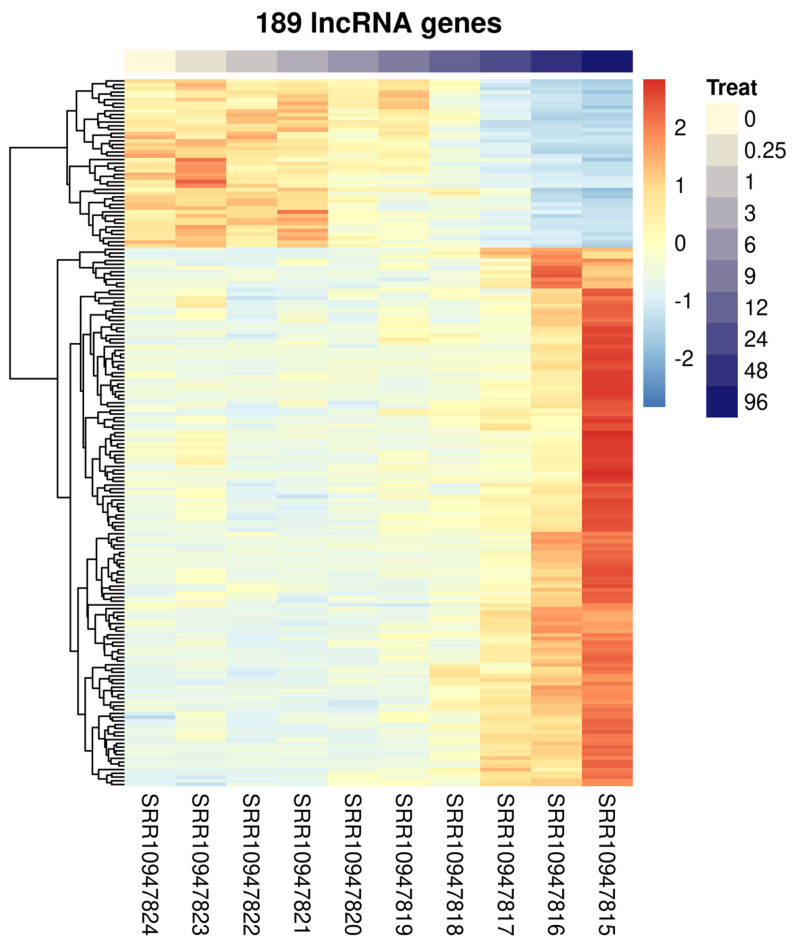
Heatmap of differentially expressed long non-coding RNAs (lncRNAs) detected by RNA-Seq of adult worm couples obtained from mice across ten time points at 0, 0.25, 1, 3, 6, 9, 12, 24, 48, and 96 h after treatment (Treat) with a single, curative dose of praziquantel (400 mg/kg) delivered by oral gavage at 7 weeks post-infection. Hierarchical clustering of differentially expressed lncRNAs (lines) from adult worm samples (columns) harvested at various time points following PZQ treatment of infected mice, as indicated by the color bar at the top and the blue color scale at right. These results were obtained by re-analyses of the RNA-Seq data from McCusker et al. [11] using the *S. mansoni* lncRNA reference transcriptome published by Maciel et al. [26]. Gene expression levels were measured by RNA-Seq and are shown as Z-scores, which are the number of standard deviations below (blue, downregulated) or above (red, upregulated) the mean expression value among treated and control samples for each gene, as indicated in the scale at right. 189 lncRNAs were considered significantly differentially expressed, being 127 long intergenic ncRNAs, 58 antisense lncRNAs and 4 sense lncRNAs (FDR < 0.05).

**Figure 2:**
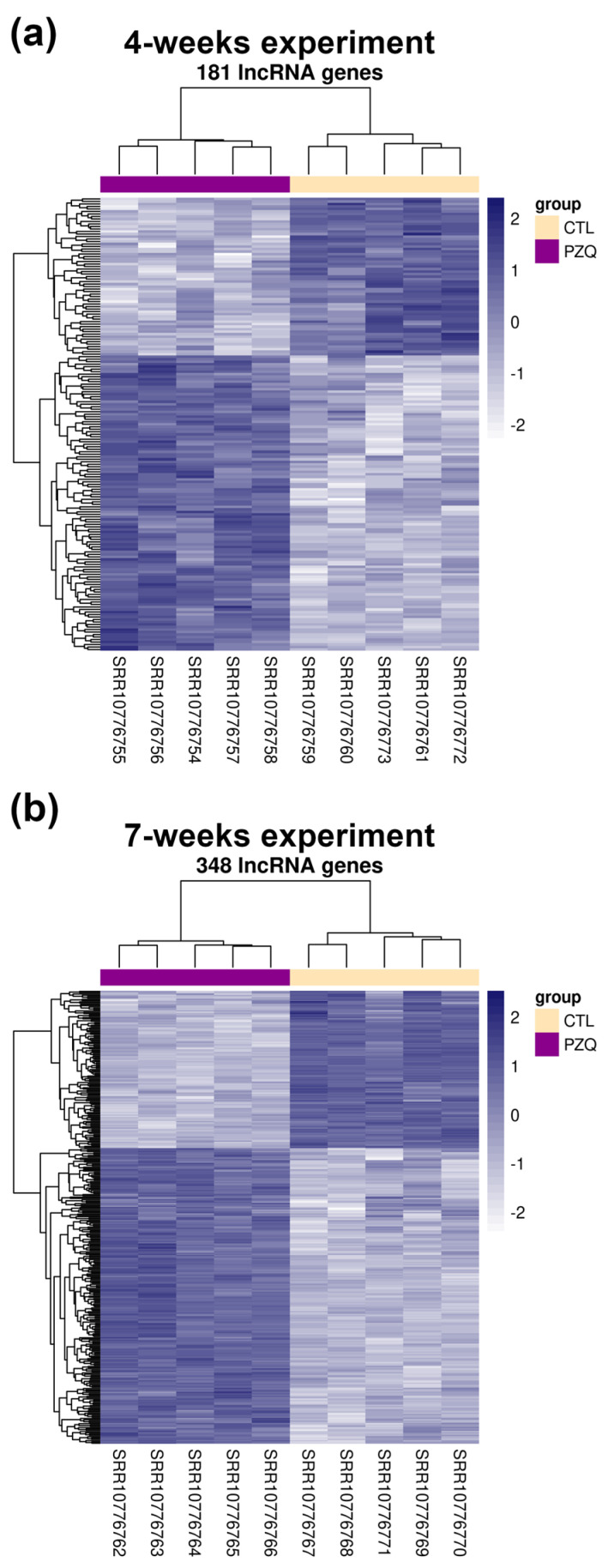
Heatmaps of differentially expressed long non-coding RNAs (lncRNAs) detected by RNA-Seq of adult worm couples obtained from mice 14 h after treatment with a single, sub-lethal dose of praziquantel (100 mg/kg) delivered by intraperitoneal injection at 4 (**a**) or 7 (**b**) weeks post-infection. Hierarchical clustering of differentially expressed lncRNAs (lines) from five adult worm sample replicates (columns) harvested 14 h following praziquantel (PZQ) treatment of infected mice or from five control (CTL) replicates, as indicated by the color bars at the top and the color legend at right. These results were obtained by re-analysis of the RNA-Seq data from McCusker et al. [11] using the *S. mansoni* lncRNA reference transcriptome published by Maciel et al. [26]. Gene expression levels were measured by RNA-Seq and are shown as Z-scores, which are the number of standard deviations below (downregulated, light blue) or above (upregulated, dark blue) the mean expression value among treated and control samples for each gene, as indicated in the scale at right. (**a**) In the 4-weeks experiment, 181 lncRNAs were considered significantly differentially expressed, being 108 long intergenic ncRNAs, 57 antisense lncRNAs and 16 sense lncRNAs (FDR < 0.05). (**b**) In the 7-weeks experiment, 348 lncRNAs were considered significantly differentially expressed, being 236 long intergenic ncRNAs, 95 antisense lncRNAs and 17 sense lncRNAs (FDR < 0.05).

In that study, McCusker et al. [11] analyzed only the protein-coding genes differentially expressed in *S. mansoni* after PZQ treatments. As lncRNA expression levels have been shown to be modulated or potentially affected by drugs in other eukaryotes [25] and also in *S. mansoni* [23], we hypothesized that lncRNA levels could be also modulated by PZQ in *S. mansoni*. Indeed, re-mapping the RNA-Seq data to the genome using a *S. mansoni* reference transcriptome previously built by us that comprises lncRNAs in addition to protein-coding genes [26], we found lncRNAs DE in *S. mansoni* worms recovered from mice treated with PZQ in all three re-analyzed RNA-Seq experiments: 189, 181 and 348 lncRNAs were found DE in the “time-course experiment” (Figure 1), in the “4-weeks experiment” (Figure 2a) and in the “7-weeks experiment” (Figure 2b), respectively. 

In the “time-course experiment” the following lncRNAs were found as DE (Figure 1 and Supplementary Table S2): 127 long intergenic non-coding RNAs (lincRNAs, being 101 upregulated and 26 downregulated), 58 antisense non-coding RNAs (SmLNCAs, being 41 upregulated and 17 downregulated) and 4 sense non-coding RNAs (SmLNCSs, being 2 upregulated and 2 downregulated). In the “4-weeks experiment”, a higher number of SmLNCSs was found as DE when compared with the “time-course experiment” (Figure 2a and Supplementary Table S3): 108 lincRNAs, being 61 upregulated and 47 downregulated, 57 SmLNCAs, being 44 upregulated and 13 downregulated and 16 SmLNCSs, being 13 upregulated and 3 downregulated. Interestingly, in the “7-weeks experiment”, the highest number of lncRNAs was found as DE (Figure 2b and Supplementary Table S4): 236 lincRNAs, being 163 upregulated and 73 downregulated, 95 SmLNCAs, being 51 upregulated and 44 downregulated and 17 SmLNCSs, being 13 upregulated and 4 downregulated.

Protein-coding genes (Smps) were also detected as DE in the “time-course experiment” (Supplementary Figure S2), in the “4-weeks experiment” (Supplementary Figure S3) and in the “7-weeks experiment” (Supplementary Figure S4). As expected, we found a good overlap in the protein-coding genes found as DE when we compared our RNA-Seq re-analyses with the analyses from McCusker et al. [11]. For the “time-course experiment”, we found in our re-analysis 760 (41%) out of the 1848 Smps previously found as DE in McCusker et al. [11]. For the “4-weeks” dataset, we found in our re-analysis 112 (83%) out of the 135 Smps previously found as DE. For the “7-weeks” dataset, we found in our re-analysis 356 (88%) out of the 405 Smps previously found as DE. These are reasonable proportions considering the differences in the reference transcriptomes used and the different read-mapping and counting software used in both analyses, with more stringent DE statistical cutoff criteria used here.

### 2.2. A Set of LncRNAs Is Concomitantly Differentially Expressed under Distinct PZQ Treatments 

**Figure 3:**
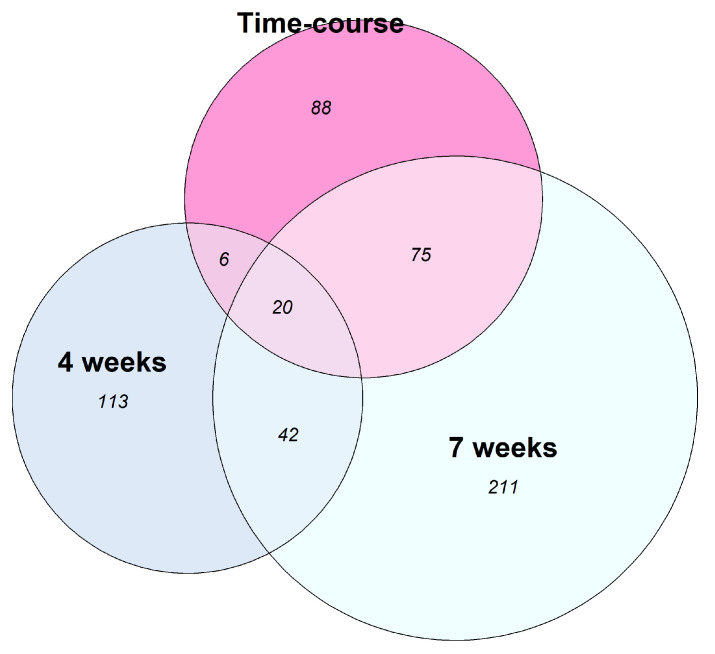
Analysis of long non-coding RNAs (lncRNAs) differentially expressed upon distinct praziquantel treatment regimens. The Venn diagram shows the number of lncRNAs that were detected as differentially expressed in one experiment alone, or at the same time in three different experiments: RNA-Seq from adult worm couples obtained from mice across ten time points at 0, 0.25, 1, 3, 6, 9, 12, 24, 48, and 96 h after treatment with a single, curative dose of PZQ (400 mg/kg) delivered by oral gavage at 7 weeks post-infection (“Time-course” experiment, pink circle); (ii) RNA-Seq from adult worm couples obtained from mice 14 h after treatment with a single, sub-lethal dose of PZQ (100 mg/kg) delivered by intraperitoneal injection at 4 weeks post-infection (“4-weeks” experiment, blue circle); (iii) RNA-Seq from adult worm couples obtained from mice 14 h after treatment with a single, sub-lethal dose of PZQ (100 mg/kg) delivered by intraperitoneal injection at 7 weeks post-infection (“7-weeks” experiment, green circle).

We evaluated whether the lncRNAs found DE are shared between the three different PZQ-treatment regimens. Interestingly, most of the lncRNAs found DE in the “time-course experiment” (101 out of 189 lncRNAs, 54%, Figure 3), are also DE in the “4-weeks” or in the “7-weeks” experiment. The overlap of 54% of lncRNAs DE in the time-course experiment with lncRNAs DE in the other treatment regimens point to a limited set of affected lncRNAs, given the total of 16,583 lncRNAs present in the reference transcriptome [26]. A higher number of DE lncRNAs is shared between the “time-course experiment” and the “7-weeks experiment” (95 out of 189, 50%) when compared with the ones shared between the “time-course experiment” and the “4-weeks experiment” (26 out of 189, 14%), and this is probably because both “time-course” and the “7-weeks” experiments were performed with adult worms. 

On the other hand, the “4-weeks experiment” shares more DE lncRNAs with the “7-weeks experiment” (62 out 181 lncRNAs) than with the “time-course experiment” (26 out 181 lncRNAs). In addition, 20 lncRNAs were found DE in all three experiments concomitantly, and we selected six of these for differential expression validation by RT-qPCR, as further explained below.

We also investigated whether the DE lncRNAs identified in our re-analysis overlapped with those modulated by 5-azacytidine [23], a drug known to affect *S. mansoni* fecundity [27]. Since 5-azacytidine is an epigenetic drug known to prevent DNA and RNA methylation [28,29], impairing the *S. mansoni* females’ transcription, translation and stem cell activities [30], we did not expect to find considerable overlap. Indeed, only a small proportion of DE lncRNAs from *in vivo* PZQ treatment experiments overlapped with those from 5-azacytidine (Supplementary Figure S5): 69 (8%) from the “4-weeks experiment”, 90 (10%) from the “7-weeks experiment”, and 59 (6%) from the “time-course experiment” out of 912 lncRNAs listed as DE after 5-azacytidine treatment. 

### 2.3. Validation by RT-qPCR of LncRNAs Differential Expression 

We selected ten candidate lncRNAs for RT-qPCR differential expression validation based on the differentially expressed genes (DEGs) identified simultaneously in the three experiments (six lncRNAs) or in the “time-course experiment” exclusively (four lncRNAs). These lncRNAs were selected based on the following criteria: (i) only lincRNAs (long intergenic non-coding RNAs) were selected, to avoid quantification of the levels of protein-coding genes expressed at the same genomic loci of antisense and sense lncRNAs and (ii) they show a wide range of expression levels in the RNA-Seq (trimmed mean of M values (TMM) from 0.10 to 172 in at least one of the conditions).

First, six protein-coding genes were used as controls for RT-qPCR differential expression validation. In the RT-qPCR assays, samples from adult worms collected after PZQ treatment of mice (400 mg/kg) at time points 0, 6, 12, 24, and 48 h post-treatment were used. Four out of the six tested protein-coding genes were validated: Smp_000660.1 (*Ornithine aminotransferase*), Smp_008660.2 (*Severin*), Smp_307450.3 (*U-actitoxin-Avd3s*) and Smp_311670.1 (*U-actitoxin-Avd3s*) showed statistically significant differential expression in at least two of the time points tested by RT-qPCR (Supplementary Figure S6, black lines). Despite the RT-qPCR results exhibiting the same expression kinetic profile observed in the RNA-Seq (Figure S6, blue lines) for Smp_132670.1 (*Myosin regulatory light chain 2 smooth muscle*) and Smp_307020.1 (*Actin-2*), these two genes did not show a statistically significant differential expression at any of the time points tested.

**Figure 4:**
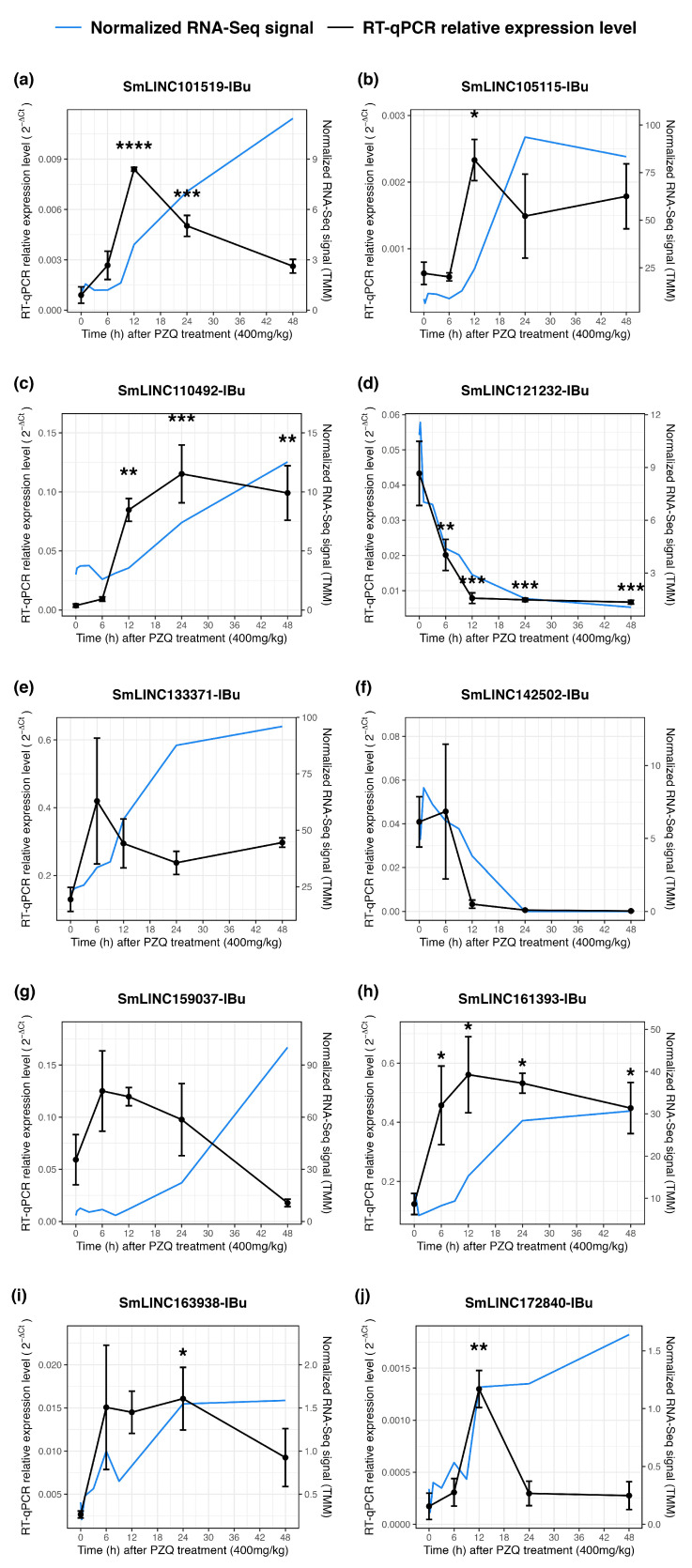
Validation by RT-qPCR of lincRNAs detected as differentially expressed upon praziquantel treatment *in vivo*. Ten lincRNAs detected by RNA-Seq as differentially expressed were selected for in vitro RT-qPCR assays, namely: (**a**) SmLINC101519-IBu, (**b**) SmLINC105115-IBu, (**c**) SmLINC110492-IBu, (**d**) SmLINC121232-IBu, (**e**) SmLINC133371-IBu, (**f**) SmLINC142502-IBu, (**g**) SmLINC159037-IBu, (**h**) SmLINC161393-IBu, (**i**) SmLINC163938-IBu, and (**j**) SmLINC172840-IBu. For each of the ten selected lincRNAs, the expression profiles obtained with RNA-Seq are shown on the right (blue line) as TMM (trimmed mean of M values), whereas the RT-qPCR results are shown on the left (black line). For the RT-qPCR data, mean ± SEM from three biological replicates is shown, and a linear mixed-effects statistical model was applied. * *p*  <  0.05, ** *p*  <  0.01, *** *p*  <  0.001, and **** *p*  <  0.0001.

We then tested by RT-qPCR, in the same samples, ten lincRNAs identified as differentially expressed in the RNA-Seq datasets. Seven of the tested lincRNAs were confirmed by RT-qPCR to show a statistically significant differential expression in at least one of the time points (Figure 4, black lines): SmLINC101519-IBu, SmLINC105115-IBu, SmLINC110492-IBu, SmLINC121232-IBu, SmLINC161393-IBu, SmLINC163938-IBu, and SmLINC172840-IBu, with changes in expression ranging from 0.003 to 81.4-fold (Figure 4, black lines). The kinetic expression profile found in the RT-qPCR assays (Figure 4f, black lines) for SmLINC142502-IBu mirrored those from the RNA-Seq (Figure 4f, blue lines), however it did not show a statistically significant differential expression in the RT-qPCR assays. For SmLINC133371-IBu (Figure 4e) and SmLINC159037-IBu (Figure 4g), distinct expression profiles were found in the RT-qPCR assays when compared with the RNA-Seq.

Overall, differential expression patterns observed in the RT-qPCR data mirrored those obtained in the RNA-Seq re-analysis: high correlations between the fold changes found in the RT-qPCR assays and in the RNA-Seq data were found for the time points 12, 24 and 48 h (Supplementary Figure S7), with Pearson correlation coefficients of 0.828 (*p*-value = 6.85 × 10^−13^), 0.829 (*p*-value = 6.08 × 10^−13^) and 0.830 (*p*-value = 5.46 × 10^−13^), respectively. To ensure the validity of our assays, non-differentially expressed lincRNAs were also tested by RT-qPCR as an additional control. As expected, the five tested lincRNAs showed no statistically significant change in expression, relative to the untreated control, throughout the time points (Supplementary Figure S8).

### 2.4. LncRNAs Modulated by Praziquantel Are Differentially Expressed along S. mansoni Life-Cycle Stages 

**Figure 5:**
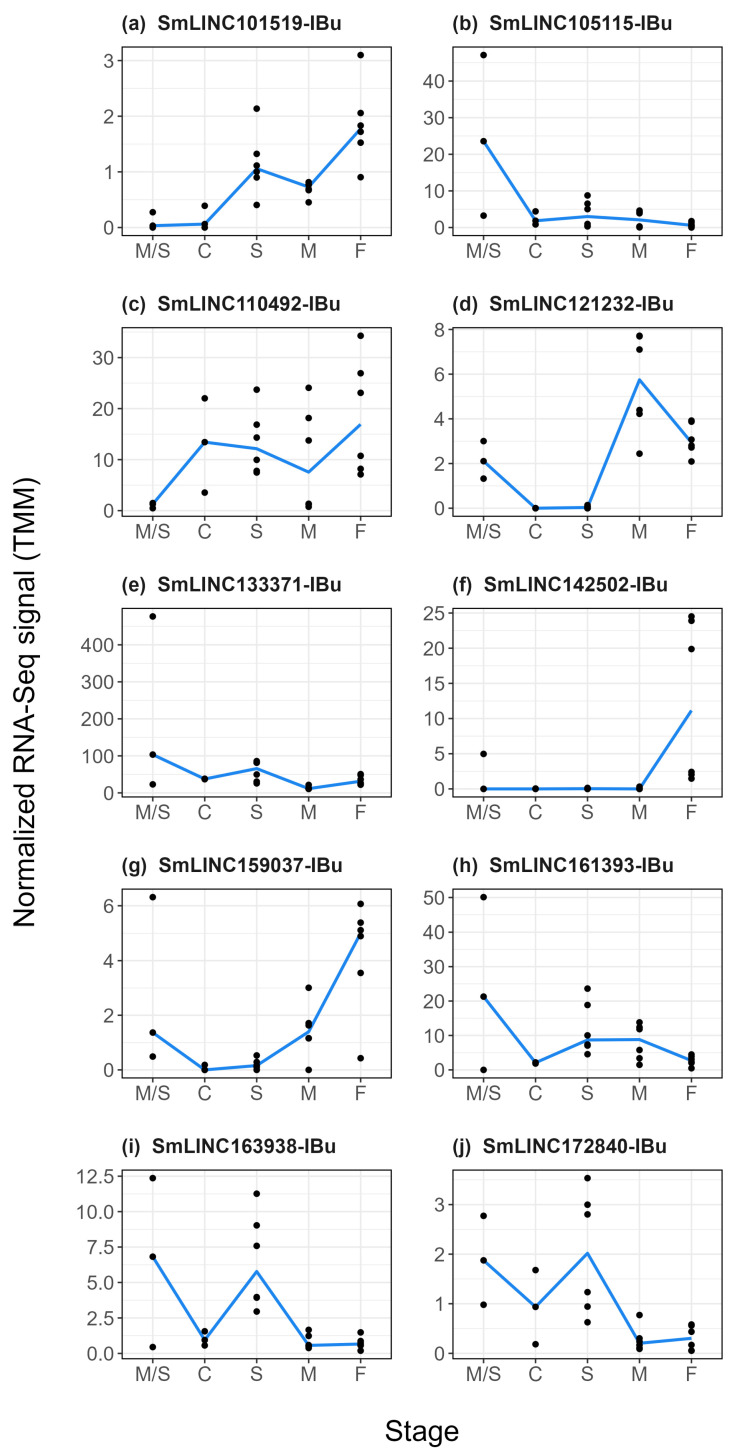
RNA-Seq expression profiles of selected lincRNAs measured in control assays at different *S. mansoni* life-cycle stages. The expression levels of the ten selected lincRNAs are shown, namely: (**a**) SmLINC101519-IBu, (**b**) SmLINC105115-IBu, (**c**) SmLINC110492-IBu, (**d**) SmLINC121232-IBu, (**e**) SmLINC133371-IBu, (**f**) SmLINC142502-IBu, (**g**) SmLINC159037-IBu, (**h**) SmLINC161393-IBu, (**i**) SmLINC163938-IBu, and (**j**) SmLINC172840-IBu. These *S. mansoni* lincRNAs were selected after re-analysis of RNA-Seq public datasets of parasites collected from mice treated with PZQ in the “time-course experiment” [11]. The y-axis shows the expression level (shown as TMM—trimmed mean of M values) for each lincRNA in control RNA-Seq assays, as compiled by Silveira et al. [31], at the stage indicated in the x-axis as follows: miracidia/sporocysts (M/S), cercariae (C), schistosomula (S), adult males (M), and adult females (F).

To evaluate whether the lncRNAs differentially expressed after PZQ treatment and tested by RT-qPCR are also expressed in other *S. mansoni* life-cycle stages, we looked at the “lncRNA transcriptome” [31] for the expression patterns of the ten selected lincRNAs in control assays. We observed a heterogeneous expression pattern distribution (Figure 5): expression of SmLINC105115-IBu (Figure 5b), SmLINC133371-IBu (Figure 5e) and SmLINC161393-IBu (Figure 5h) are enriched in miracidia and sporocysts stages, whereas SmLINC163938-IBu (Figure 5i) and SmLINC172840-IBu (Figure 5j) have higher expression levels in schistosomula.

While SmLINC121232-IBu shows higher expression levels in adult males (Figure 5d), SmLINC101519-IBu (Figure 5a), SmLINC142502-IBu (Figure 5f) and SmLINC159037-IBu (Figure 5g) are enriched in adult females, with SmLINC142502-IBu being exclusively expressed in this life-cycle stage. In turn, SmLINC110492-IBu (Figure 5c) is broadly expressed in cercariae, schistosomula, adult males and adult females. These results show that most of the tested lincRNAs are not specific to a single stage and may play roles in different *S. mansoni* life-cycle stages. As expected, most of the protein-coding genes were found not to be stage-specific, with only Smp_132670.1 and Smp_307020.1 showing enrichment in adult males (Supplementary Figure S9). 

### 2.5. Weighted Gene Co-Expression Network Analysis Shows Terms Related to Drug Response Mechanisms 

We performed a weighted gene co-expression network analysis (WGCNA) [32] on the RNA-Seq data from all three experiments together to identify co-expression networks integrating gene expression differences following *in vivo* PZQ treatment. After counts normalization and filtering, 14,040 transcripts remained (11,796 protein-coding genes and 2244 lncRNAs), and one sample (SRR10947815; “PZQ 96hr”), out of 30, was removed from the analysis. A total of 15 co-expression modules were identified (Supplementary Figure S10 and Supplementary Table S5).

We found that, except for SmLINC142502-IBu, all DE lncRNAs tested in RT-qPCR are part of four different modules, namely “brown2” (SmLINC163938-IBu), “floralwhite” (SmLINC101519-IBu, SmLINC105115-IBu, SmLINC110492-IBu, SmLINC133371-IBu and SmLINC159037-IBu), “plum” (SmLINC161393-IBu), and “plum2” (SmLINC121232-IBu and SmLINC172840-IBu). These lincRNAs show a high and significant membership to their corresponding modules (Supplementary Figure S11).

**Figure 6:**
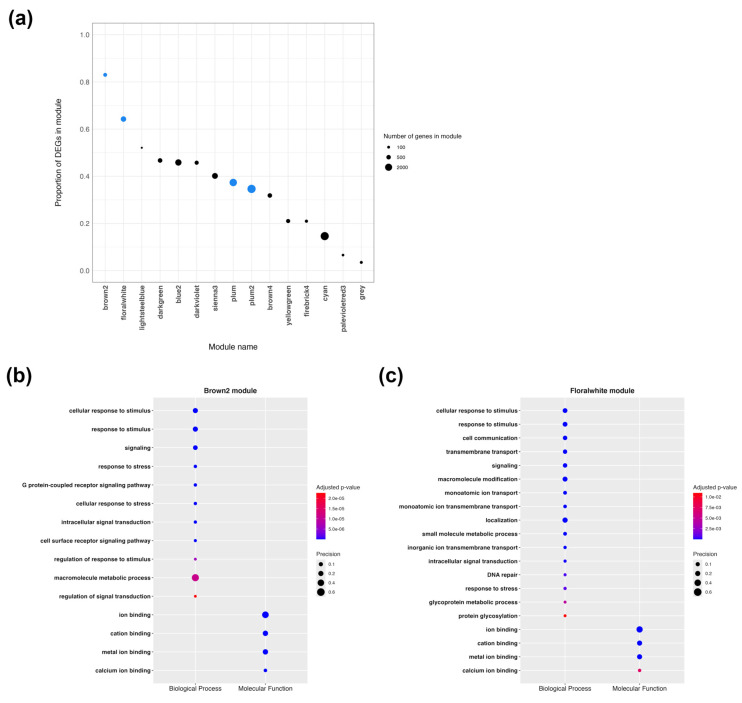
Analysis of the co-expression modules identified in WGCNA. (**a**) This plot shows the proportion of differentially expressed genes (DEGs) inside each of the 15 modules. The total number of genes inside each module is proportional to the point size; blue points (representing “brown2”, “floralwhite”, “plum” and “plum2”) indicate modules that contain the differentially expressed lncRNAs tested by RT-qPCR. The most significantly enriched and relevant gene ontology (GO) terms, including biological process and molecular function terms, are shown for “brown2” (**b**) and “floralwhite” (**c**) modules. The size of each point is proportional to the precision, also known as gene ratio (i.e., the proportion of genes in the input list that are annotated to the function). The colors show the statistical significance of the enrichment.

In addition to containing the lincRNAs tested by RT-qPCR, these four modules also stand out for other reasons. Two of them show the highest number of DEGs among all modules (Figure 6a), making them the most enriched in DEGs: in the “brown2” module, 244 out of 294 genes are DE (83%; *p*-value = 7.32 × 10^−65^, Fisher’s Exact Test), and in the “floralwhite” module, 584 out of 909 genes are DE (64%; *p*-value = 1.97 × 10^−75^, Fisher’s Exact Test). Although “plum” and “plum2” modules also contain a considerable amount of DEGs, only “plum” is significantly enriched, with 837 out of 2241 genes being DE (37%; *p*-value = 1.40 × 10^−2^). On the other hand, “plum” and “plum2” modules exhibit, respectively, the highest association with PZQ treatment (*R*^2^ = 0.53, *p*-value = 7.34 × 10^−6^; and *R*^2^ = 0.52, *p*-value = 1.13 × 10^−5^). As a result, we observe a clear separation of PZQ-treated samples and control group samples into two distinct clusters, distributed in the plot area of these modules’ eigengenes (the first principal component, PC1, of the expression matrix of the corresponding module) (Supplementary Figure S12).

Functional enrichment analysis of the protein-coding genes inside these four modules reveals that they are enriched with gene ontology (GO) terms that might indicate drug response mechanisms (Figure 6b,c and Supplementary Figure S13). For example, the “brown2” module is most significantly enriched in biological processes (BPs) such as “response to stimulus”, “signaling”, “response to stress”, and “cell surface receptor signaling pathway” (Figure 6b). The “floralwhite” module is also enriched in “response to stimulus”, “signaling” and “response to stress”; in addition, BP terms such as “transmembrane transport”, “monoatomic ion (transmembrane) transport”, “small molecule metabolic process”, and “DNA repair” are noteworthy (Figure 6c). Although in “plum” and “plum2” modules most of the enriched terms are related to metabolism, they also show enrichment among the terms as found in “brown2” and “floralwhite” modules (Supplementary Figure S13). Regarding molecular function GO terms, these four modules show significant enrichment in relevant terms such as “metal ion binding”, “calcium ion binding”, and “small molecule binding”.

Remarkably, 31 out of the 37 protein-coding genes highlighted by McCusker et al. [11] as involved in PZQ response are assigned to eight of the modules found here. Importantly, the majority of the highlighted Smps (25 Smps, not including isoforms) belong to the four most prominent modules: 12 Smps are in the “floralwhite” module, 11 Smps are in the “brown2” module, 2 Smps are in the “plum” module, and 4 Smps are in the “plum2” module.

### 2.6. PZQ-Modulated lncRNAs Co-Localize with PZQ-Modulated Smps in Different Cell Types

**Figure 7:**
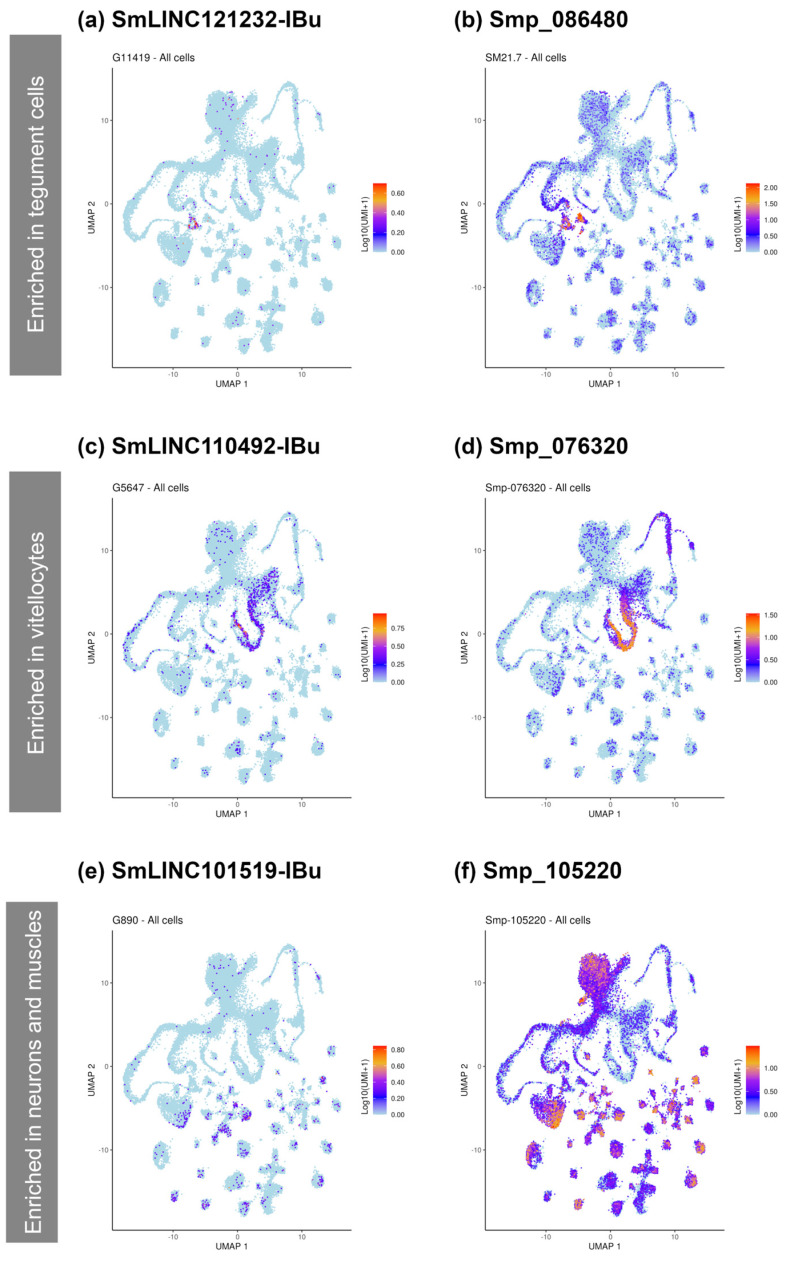
Single-cell clusters expression profiles of three RT-qPCR validated differentially expressed lncRNAs matching the expression profile of three differentially expressed protein-coding genes modulated under praziquantel exposure [11]. UMAP plots show the expression enrichment of genes and are colored by gene expression (blue = low, red = high). The scale represents log10(UMIs+1). The genes with expression enrichment in tegument are (**a**) SmLINC121232-IBu and (**b**) Smp_086480 (SmTAL2); in vitellocytes (**c**) SmLINC110492-IBu and (**d**) Smp_076320 (Myb/SANT-like DNA-binding domain-containing protein); and in neurons and muscles (**e**) SmLINC101519-IBu and (**f**) Smp_105220 (Lymphocyte antigen 6B).

To identify the cell types in which the RT-qPCR validated differentially expressed lncRNAs under PZQ treatment are mainly expressed, we searched their expression localization on the *Schistosoma mansoni* single-cell cluster atlas [33]. Then, we matched these results with protein-coding genes modulated by PZQ that had been highlighted by McCusker et al. [11], which are expressed in the same single-cell clusters (Figure 7).

The most prominent match we identified was SmLINC121232-IBu (Figure 7a), which co-localizes with Smp_086480 (*SmTAL2*), Smp_086530 (*SmTAL3*), and Smp_169200 (*SmTAL11*), at the tegument cell cluster (Figure 7a, b). All these transcripts were found to be downregulated in our analysis and are highlighted DEGs in McCusker et al. [11] due to the immunomodulatory activity of the tegument allergen-like proteins (SmTALs) they encode [34,35,36].

Additionally, two other matching genes were found: Smp_076320 (*Myb/SANT-like DNA-binding domain-containing protein 3*) (Figure 7d); which is significantly enriched in vitellocytes, matching the cell cluster expression profile of SmLINC110492-IBu (Figure 7c); and the Smp_105220 (*Lymphocyte antigen 6B*, or *SmLy6B*) (Figure 7f) which is significantly enriched in neurons and muscles cell clusters, matching the profile of SmLINC101519-IBu (Figure 7e).

## 3. Discussion

Here, we have shown that *S. mansoni* long non-coding RNA levels can be modulated *in vivo* by praziquantel (PZQ), the only drug currently used to treat schistosomiasis. Hundreds of lncRNAs were found to be differentially expressed (DE) in *S. mansoni* after PZQ exposure of mice infected for 4 or 7 weeks. This shows that there is an extensive reprogramming of the gene expression in *S. mansoni* worms that goes beyond the changes in protein-coding gene expression. Indeed, this observation is in line with previous studies showing that lncRNA levels can be modulated by drugs in *S. mansoni* [23] and in other eukaryotes [25,37,38,39,40].

Several studies have demonstrated and validated the modulation of lncRNA levels in other eukaryotes as a response to different drugs [25,37,38,39,40]. According to the recently published “ncRNADrug” database, a manually curated resource containing thousands of ncRNAs in current literature found to be associated with drug response and resistance [37], a set of 67 unique lncRNAs has been validated as DE under the regulation of 74 different molecules, whilst regulating the expression of 127 unique genes.

In this regard, we sought to investigate possible roles of the lncRNAs that we validated as DE, and we observed that genes found to be modulated *in vivo* by PZQ are grouped into 15 co-expression modules, four of which contain the selected DE lncRNAs and most of the DE protein-coding genes (Smps) found by McCusker et al. [11]. Being co-expressed in the same modules suggests that these lncRNAs are potential regulators of the protein-coding genes whose expression levels are also modulated by PZQ. 

We also observed that some of the PZQ-modulated Smps co-localize at the same single-cell clusters with the RT-qPCR-validated DE lncRNAs. For instance, Smp_086480 (SmTAL2), a member of the *Schistosoma mansoni* tegument allergen-like protein family (SmTAL) co-localizes with SmLINC121232-IBu. It is described that the majority of known SmTALs function as calcium-ion binding proteins, an important step of calcium signaling in eukaryotic cells [34,35,36]. Previous studies have successfully demonstrated that, except for SmTAL3 and SmTAL5, the other 11 known SmTALs bind to calcium-ions [34,35]. Importantly, SmTAL1, 4, 5, and 8 were shown to interact with PZQ [35] and, therefore, these proteins may be involved in the worm’s response to PZQ along with SmLINC121232-IBu.

In addition, we observed that SmLINC110492-IBu co-localizes with the vitellaria-specific Smp_076320, a *Myb/SANT-like DNA-binding domain-containing protein*. These DNA-binding domains are associated with transcription factors, chromatin-remodeling proteins and other transcriptional regulation proteins [41,42,43]. Interestingly, an orthologue of this protein was recently identified in the trematode *Fasciola hepatica* as a candidate gene involved in drug resistance [44]. 

Finally, it is also worth mentioning that SmLINC101519-IBu co-localizes with the muscle- and neuron-enriched Smp_105220 (*Lymphocyte antigen 6B*, *SmLy6B*). It is believed that the Ly6 protein family is tegument surface specific and capable of inducing host immune responses [45,46], but it has also been shown that in adult worms their expression is up-regulated in intra-parasite tissues, including parenchyma and muscles [47]. In humans and other mammals these proteins are involved in cell proliferation, migration, cell–cell interaction, cell signaling, and in the immune response [47,48,49].

Interestingly, all of these genes are expressed in the four most prominent co-expression modules in terms of the proportion of differentially expressed genes, correlation to PZQ treatment, and the presence of the validated lncRNAs: “brown2”, “floralwhite”, “plum”, and “plum2”. On top of that, these four co-expression modules are enriched in the calcium-ion binding function, as well as other processes that may be related to PZQ response, such as transmembrane ion transport, response to stimulus and stress, small molecule metabolism, and DNA repair.

The fact that some of the validated differentially expressed lncRNAs co-localize with these protein-coding genes in the same cell cluster and co-expression modules, especially SmLINC101519-IBu and Smp_105220, which are both part of the “floralwhite” module, raises the hypothesis of a possible regulation of these protein-coding genes by the lncRNAs identified here, and regulation of these specific proteins may be coherent in response to PZQ treatment. The SmTAL products could be related to the classic well-studied PZQ response in *S. mansoni* [34,35]. Further, although there is uncertainty around the functions of Myb/SANT-like domain-containing protein and SmLy6B, one might speculate they could be associated to stress response due to PZQ treatment. This is in line with the vitellaria-specific Smp_076320 being down-regulated, and the Smp_105220 being up-regulated upon PZQ treatment.

The molecular mechanism of action of PZQ leading to worm death is still poorly understood, although the drug has been the main anthelmintic used to control schistosomiasis for over 40 years [5,6,8]. It has been shown that PZQ has deleterious effects on parasite musculature and tegument in vitro [50,51], however the effects of PZQ treatments *in vivo* were not clear until recently, given the short half-life of PZQ [52] and the importance of host immune system engagement for drug efficacy in animal models [53,54]. Recently, McCusker et al. [11] showed that worms harvested from mouse livers following sub-lethal PZQ treatment revealed drug-evoked changes in the expression of putative immunomodulatory and anticoagulant gene products, such as tegument-like allergens [35] and Kunitz-type protease inhibitors [55], which can reflect mechanisms of parasite immune-evasion in response to chemotherapy [11]. Since lncRNAs have been shown to act as regulators of the expression of protein-coding genes through different mechanisms [12,13], it is possible that some of the lncRNAs found here by re-analysis of these RNA-Seq datasets [11] as differentially expressed upon *in vivo* PZQ treatment may act by regulating the expression of these putative immunomodulatory and anticoagulant protein-coding genes [11].

Moreover, several lncRNAs have been implied in drug resistance mechanisms in other organisms, in particular human cancers, where their mechanisms of action in response to drug treatment are well studied. Several lncRNAs have been reported in association with increase of drug efflux and metabolism, inhibition of apoptosis, induced DNA repair, and protection from oxidative stress [16,56,57]. In this context, it is crucial to note that drug resistance in *S. mansoni* is a growing concern in public health, since the emergence of resistant strains has already been observed in laboratory and is particularly concerning in the field [5,6,8]. It is possible that similar lncRNA-associated drug resistance mechanisms described in other models are related to the *S. mansoni* lncRNAs found here to be regulated by PZQ.

There are several mechanisms by which lncRNA expression can be regulated, and that might underlie the drug induced changes in lncRNA expression. In general, similar to that of protein-coding genes, lncRNA expression regulation is determined by modulation of chromatin state and by post-transcriptional modifications [58,59]. The primary mechanism for the regulation of lncRNA expression is based on chromatin state. LncRNAs might be either down-regulated by DNA hypermethylation or up-regulated by histone deacetylation in promoter regions. They usually have low CpG promoters, which is indicative of DNA hypermethylation throughout evolutionary history, and are thus associated with fewer transcriptional activation histone marks (e.g., H3K4me3) resulting in lower expression levels. Additionally, many transcription factors acting upon lncRNAs are known to be shared with protein-coding genes, and finer regulation of promoter activity operates in association with microRNAs [58]. Post-transcriptional regulation impacting RNA stability is also important for their regulation. For example, there are more than 100 distinct modified nucleotides in lncRNAs [58]. It is also known that microRNA interaction with RNA-binding proteins (RBP) mediates decay of lncRNA [58,59], as well as enzymes that control decapping, deadenylation, and nucleolytic activity [59].

Unlike short RNAs [60,61,62,63,64,65,66], the mechanisms of regulation of lncRNAs are largely unknown in parasites. In addition, while short RNAs (especially microRNAs) have been more explored in various helminths [67,68,69,70], lncRNAs have received little attention, being identified by transcriptomic approaches only in a few helminths other than *S. mansoni* and in protozoans [19,71,72,73] or studied in a limited number of free-living nematodes [74,75]. Therefore, it is time to consider lncRNAs as regulated by or as possible drug targets also in parasitic diseases, especially because they show lower conservation in their primary sequences between species than protein-coding genes [12,13], which in principle would favor the development of therapeutical strategies with less adverse effects [20]. 

The choice of lncRNAs to be further validated as drug targets should be based on the appropriate selection of lncRNA candidates, as reviewed by Silveira et al. [19]. This selection should be guided by functional characterization of the lncRNA as well as by the demonstration of the lncRNA relevance to the parasite biology. We have recently shown that lncRNAs are key components intervening in *S. mansoni* adult worm homeostasis, being essential for adult worm pairing status and survival in the mammalian host [24]. Interestingly, two of the lncRNAs modulated by PZQ, found here, are also differentially expressed between paired and unpaired *S. mansoni* adult worms (SmLINC133371-IBu and SmLINC101519-IBu), highlighting their involvement in adult worm homeostasis. 

In summary, this study adds a deeper layer on the understanding of the effects of PZQ in *S. mansoni* and sheds light on the relevance of looking at lncRNA regulation in response to drug treatment in parasites. These data are, to our knowledge, the first report of modulation of lncRNA levels by a drug *in vivo* in any helminth. The mechanisms involving lncRNAs and protein-coding gene expression networks, to be further explored, will provide insights into potential mechanisms for PZQ treatment failure or routes to anthelmintic drug resistance.

## 4. Materials and Methods

### 4.1. Re-Analyses of PZQ Treatment RNA-Seq Data

Public RNA-Seq data previously generated by McCusker et al. [11] from *Schistosoma mansoni* worms after *in vivo* PZQ treatment were downloaded from the SRA-NCBI database (PRJNA597909 and PRJNA602528). Libraries were previously generated using the TruSeq Stranded mRNA kit (Illumina, San Diego, CA, USA) and sequenced using the Illumina HiSeq 2500 system [11]. The datasets included both a time-course and a transcriptional response experiment involving adult and immature worms. For the time-course experiments, adult worms were harvested from mice (*n* = 4 per time point) at ten different time points (0, 0.25, 1, 3, 6, 9, 12, 24, 48, and 96 h) after the administration to mice of a single curative dose of PZQ (400 mg/kg) 7 weeks post-infection. The transcriptional response experiments were performed by harvesting worms 14 h after treating mice (*n* = 5 per group) with either vehicle control or a sub-lethal PZQ dose (100 mg/kg). These transcriptional response experiments were conducted on mice treated with PZQ at 4 weeks post-infection (yielding juvenile worms) or at 7 weeks post-infection (yielding adult worms).

As previously established by our group [23,24,31,76], the identification and differential expression analysis of lncRNAs and protein-coding genes were performed using the following pipeline: primarily, adapters and low-quality reads were filtered out using fastqc v 0.11.9 [77] and fastp v 0.20.0 [78]. The *S. mansoni* genome sequence v.7 (PRJEA36577) from the WormBase ParaSite resource (WBPS14), along with the lncRNA transcriptome identified and annotated by Maciel et al. [26], which comprises 16,583 lncRNA genes in addition to 12,693 protein-coding genes, were used as references. STAR v 2.7.3a [79] was used to align the reads, which were quantified using RSEM v 1.3.1 [80]. The counts were normalized with the TMM method using edgeR v 3.36.0 [81,82,83]. Two distinct approaches were considered for statistical analysis of differential expression: limma+voom [84] and svaseq+edgeR [81,85]. Only the genes with an FDR lower than 0.05 in both analyses were considered differentially expressed (DE). For the DE genes (DEGs) in the time-course experiment, fold-change was estimated by a generalized linear model.

### 4.2. Selection of LncRNAs for RT-qPCR Validation

We selected ten candidate lncRNAs for RT-qPCR differential expression validation based on the DEGs identified within all three experiments or in the time-course experiment exclusively. Six candidate protein-coding genes were also selected for RT-qPCR differential expression validation. Additionally, the five most stable non-DE protein-coding genes across all time points were chosen as candidate reference genes for the RT-qPCR assays, given the better suitability of condition-specific reference genes as normalizers over standard housekeeping genes [31,86]. NormFinder [87] and geNorm [88] software were employed, via the online tool RefFinder [89] (https://blooge.cn/RefFinder/, accessed on 9 January 2024), to determine the two most stable candidate reference genes (Smp_017650.1 and Smp_036130.1), which were used to normalize the Ct values of the targeted genes and calculate their relative expression (2^−∆∆Ct^).

### 4.3. RNA Extraction, Quantification, and Quality Assessment

To validate the findings from our RNA-Seq re-analyses, we used worms obtained in a time-course experiment, performed by McCusker et al. [11] to validate protein-coding genes DE by RT-qPCR assays [11]. This experiment employed the same treatment as previously described for the RNA-Seq experiments, and a similar set of time points after PZQ treatment (0, 6, 12, 24, and 48 h, with *n* = 6 biological replicates each). 

Total RNA was extracted using a combination of TRIzol (Invitrogen, Carlsbad, CA, USA) and the RNeasy Micro Kit (Qiagen, Waltham, MA, USA) for purification, according to the manufacturer’s instructions. RNA samples were quantified using the Qubit RNA HS Assay Kit (Thermo Fisher Scientific) in a Qubit 2.0 Fluorometer (Thermo Fisher Scientific) and their integrities were verified in a 2100 Bioanalyzer Instrument (Agilent Technologies, Santa Clara, CA, USA) with Agilent RNA 6000 Pico Kit (Agilent Technologies).

### 4.4. Primer Design, Reverse Transcription, and Quantitative PCR (RT-qPCR) Assays

All primer pairs were designed using Primer3 (https://bioinfo.ut.ee/primer3-0.4.0/, accessed on 9 January 2024), aiming for primer pairs designed between two exons whenever possible. Each primer was designed with the following parameters: maximum primer size of 25 nt, annealing temperature ranging from 57 °C to 63 °C, GC content between 30% and 60%, product size varying between 90 and 250 nt, and a maximum allowable length of a mononucleotide repeat set to three. The primer sequences and efficiencies are shown in Supplementary Table S6.

We used Superscript IV (Life Technologies, Carlsbad, CA, USA) for the reverse transcription, generating cDNA which was diluted eight times in water. The RT-qPCR assays were performed using 1X LightCycler 480 SYBR Green I Master Mix (Roche Diagnostics, Basel, Switzerland) with 800 nM of each oligonucleotide pair on a LightCycler 480 System instrument (Roche Diagnostics). All primers were tested in technical triplicates for all samples, in addition to a no template control (NTC).

To calculate differential gene expression, the delta-Ct method was applied [90]. Raw Ct values are shown in Supplementary Table S7. Differences in the expression of the assessed genes across all five time points were evaluated by a linear mixed-effects model [91]. This type of model accounts for the random effects observed in our samples, leading to a more accurate fit [91]. The model fitting and determination of significance (at a 0.05 significance level) were performed with the R package nlme v 3.1-164 [92].

### 4.5. Gene Expression Patterns of Selected Genes across S. mansoni Life-Cycle Stages

We generated plots showcasing the expression patterns for the selected lncRNAs and protein-coding genes across *S. mansoni* life-cycle stages (miracidium, sporocyst, cercaria, schistosomulum, adult male, and adult female). These plots were made using publicly available RNA-Seq normalized counts (TMM), from Silveira et al. [31], available at https://verjolab.shinyapps.io/Reference-genes/ (accessed on 9 January 2024). 

### 4.6. Weighted Gene Co-Expression Network Analysis (WGCNA)

We performed a WGCNA on the RNA-Seq data from all the three *in vivo* PZQ experiments together, using the R package WGCNA v1.72.5 [32]. First, the transcripts count for the “time-course experiment”, “4-weeks experiment”, and “7-weeks experiment” were normalized applying a variance stabilizing transformation with DESeq2 v1.38.3 [93]. We considered the genes expressed only if their counts were greater than or equal to three in at least 75% of the samples. The sample SRR10947815 was removed from this analysis, because of its outlier behavior observed in the hierarchical clustering, which isolated it from any sample cluster.

To maximize scale-free topology properties, fit index was calculated as a function of a series of soft-threshold powers, in order to pick the best power (Figure S10a). Based on fit index (*R*^2^) and mean connectivity (k), power equal to 9 (*R*^2^ = 0.973, *k* = 86.36) was selected for network construction and module detection. Modules were constructed based on topological overlap matrix (TOM) dissimilarity (1 − TOM). The closest modules were then merged based on their eigengenes values (the first principal component, PC1, of the expression matrix of the corresponding module) dissimilarity distance, considering a 0.40 dissimilarity threshold (Supplementary Figure S10b).

Intramodular analysis was carried out on the merged modules to identify genes’ membership, i.e., the Pearson’s correlation between gene expression data and their corresponding module eigengene (Supplementary Figure S11). To assess whether the modules were enriched in DEGs, a one-tailed Fisher’s exact test was conducted, with the alternative hypothesis stating that the observed number of DEGs in a module is greater than expected. Furthermore, each module eigengene value was investigated with sample group (PZQ-treated or control) in a linear model (eigengene ~ sample group) in order to evaluate the association between modules and PZQ treatment.

Functional profiling of the protein-coding genes inside the modules was performed with gProfiler2 v0.2.3 [94] with g:SCS multiple testing correction method. Gene ontology (GO) terms annotation of *S. mansoni* protein-coding genes (Smp) were obtained using gProfiler’s “Smansoni_v7” database. REVIGO v.1.8.1 [95] was used to remove redundancy in enriched GO terms (Figure 6 and Supplementary Figure S13).

### 4.7. Single-Cell Clusters Search of LncRNAs and Protein-Coding Genes

The *S. mansoni* single-cell clusters atlas publicly available at *Schistosoma mansoni* LncRNAs Database (http://verjolab.usp.br:8081/ accessed on 6 March 2024) [33] was used to identify the cell type where each validated DE lncRNA, and protein-coding gene highlighted by McCusker et al. [11], is expressed and significantly enriched. Results of matching cell types between the lncRNA and the protein-coding genes were selected.

## Data Availability

All data generated or analyzed during this study are included in this published article (and its Supplementary Information files).

## Supplementary Materials

The following supporting information can be downloaded at: https://www.mdpi.com/article/10.3390/ncrna10020027/s1; Table S1: RNA-Seq samples analyzed and alignment statistics; Table S2: List of transcripts differentially expressed from *S. mansoni* adult worm couples obtained across 10 timepoints of 0, 0.25, 1, 3, 6, 9, 12, 24, 48 and 96 h from mice after treatment with a single, curative dose of PZQ (400 mg/kg) delivered by oral gavage at 7 weeks post-infection (PRJNA602528); Table S3: List of transcripts differentially expressed from *S. mansoni* adult worm couples obtained from mice 14 hours after treatment with a single, sub-lethal dose of PZQ (100 mg/kg) delivered by intraperitoneal injection at 4 weeks post-infection (PRJNA597909); Table S4: List of transcripts differentially expressed from *S. mansoni* adult worm couples obtained from mice 14 hours after treatment with a single, sub-lethal dose of PZQ (100 mg/kg) delivered by intraperitoneal injection at 7 weeks post-infection (PRJNA597909); Table S5: List of genes belonging to each module according to the WGCNA analysis; Table S6: List of primers used in RT-qPCR; Table S7: Cts (Cycle Threshold) of the lncRNAs and Smps obtained in the RT-qPCR assays; Figure S1: Clustering of RNA-Seq biological replicates assessed by principal 12 component analysis (PCA). Figure S2: Heatmap of differentially expressed protein-coding genes (Smps) detected by RNA-Seq of adult worm couples obtained from mice across 10 timepoints at 0, 0.25, 1, 3, 6, 9, 12, 24, 48 and 96 h after treatment (Treat) with a single, curative dose of PZQ (400 mg/kg) delivered by oral gavage at 7 weeks post-infection; Figure S3: Heatmaps of differentially expressed protein-coding genes (Smps) detected by RNA-Seq of adult worm couples obtained from mice 14 h after treatment with a single, sub-lethal dose of PZQ (100 mg/kg) delivered by intraperitoneal injection at 4 weeks post-infection; Figure S4: Heatmaps of differentially expressed protein-coding genes (Smps) detected by RNA-Seq of adult worm couples obtained from mice 14 h after treatment with a single, sub-lethal dose of PZQ (100 mg/kg) delivered by intraperitoneal injection at 7 weeks post-infection; Figure S5: Analysis of long non-coding RNAs (lncRNAs) differentially expressed upon distinct praziquantel (PZQ) treatment regimens and 5-azacytidine (5-AzaC) treatment; Figure S6: Validation by RT-qPCR of protein-coding genes (Smps) detected as differentially expressed upon praziquantel treatment *in vivo*; Figure S7: Correlation between RNA-Seq and RT-qPCR analyses; Figure S8: RT-qPCR results of long non-coding RNAs (lncRNAs) not detected as differentially expressed upon praziquantel treatment *in vivo*; Figure S9: RNA-Seq expression profiles of selected protein-coding genes (Smps) measured in control assays at different *S. mansoni* life-cycle stages; Figure S10: Weighted Gene Co-Expression Network Analysis (WGCNA) parameters and results for the RNA-Seq experiments data; Figure S11: Gene-Module correlation for the four modules containing the differentially expressed long non-coding RNAs (lncRNAs) tested by RT-qPCR; Figure S12: Clustering of RNA-Seq biological replicates assessed by modules eigengenes values; Figure S13: Functional profiling of the protein-coding genes inside “plum” and “plum2” modules.
