# Supplementary material for: Long Non-Coding RNA Levels Are Modulated in Schistosoma mansoni following In Vivo Praziquantel Exposure
Source: Noncoding RNA. 2024 Apr 19;10(2):27. doi: 10.3390/ncrna10020027 (PMC11053911; doi:10.3390/ncrna10020027)
Supplement: Supplementary file 1 [file ncrna-10-00027-s001.zip › Proof-Supp-Ms-Poli.pdf]

Article

Long Non-Coding RNA Levels Are Modulated in *Schistosoma mansoni* following *In Vivo* Praziquantel Exposure

Pedro Jardim Poli[1], Ágatha Fischer-Carvalho[1], Ana Carolina Tahira[1], John D. Chan[2], Sergio Verjovski-Almeida[1,3], Murilo Sena Amaral[1*]

[1] Laboratório de Ciclo Celular, Instituto Butantan, 05503-900, São Paulo, SP, Brazil
[2] University of Wisconsin - Oshkosh, Oshkosh, WI, USA
[3] Instituto de Química, Universidade de São Paulo, 05508-900, São Paulo, SP, Brazil
* Correspondence: murilo.amaral@butantan.gov.br; Tel:+551126273855

Supplementary Materials

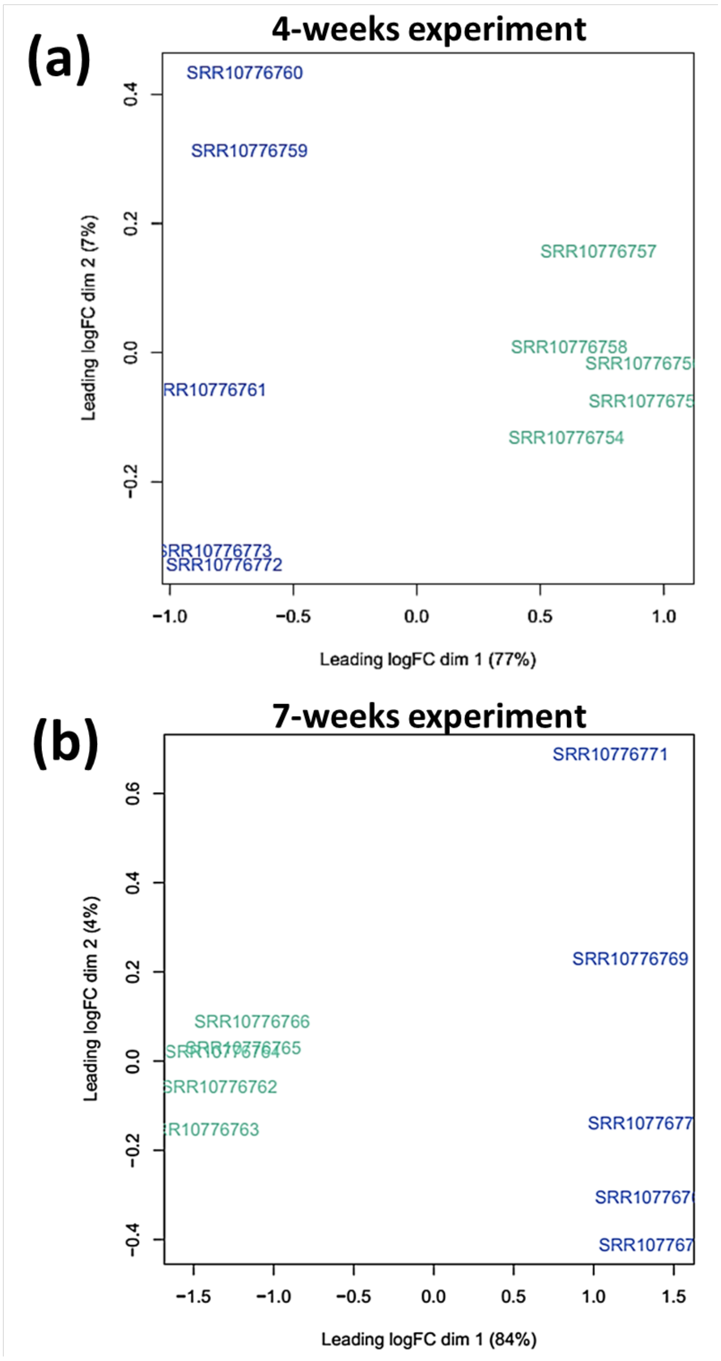

Supplementary Figure S1. Clustering of RNA-Seq biological replicates assessed by principal component analysis (PCA). RNA-Seq data from McCusker et al. [1] were re-analyzed using the *S. mansoni* genome PRJEA36577 (v.7) retrieved from WormBase and a published reference transcriptome [2] that also includes long non-coding RNAs, besides protein-coding genes. The RNA-Seq datasets comprise samples from adult worm couples obtained from mice 14 h after treatment with a single, sub-lethal dose of PZQ (100 mg/kg) delivered by intraperitoneal injection at 4 (**a**) or 7 (**b**) weeks post-infection. PCA plot was obtained after normalization using the vst function followed by the plotPCA function from DESeq2. Samples are represented by their SRA codes in five biological replicates, which are separated by their first two principal components: control samples are shown in blue, and the PZQ-treated samples are shown in green.

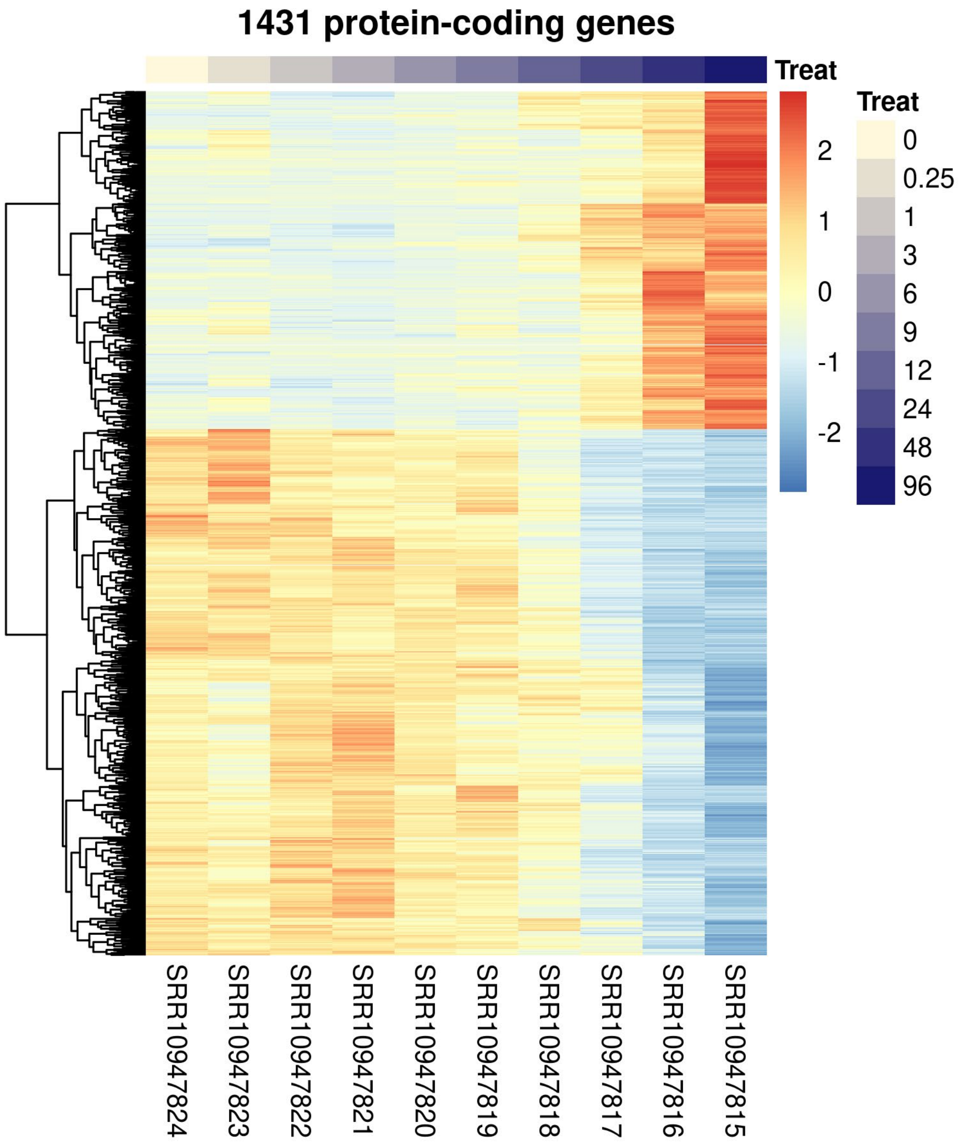

Supplementary Figure S2. Heatmap of differentially expressed protein-coding genes (Smps) detected by RNA-seq of adult worm couples obtained from mice across 10 timepoints at 0, 0.25, 1, 3, 6, 9, 12, 24, 48 and 96 h after treatment (Treat) with a single, curative dose of PZQ (400 mg/kg) delivered by oral gavage at 7 weeks post-infection. Hierarchical clustering of differentially expressed Smps (lines) in 5 adult worm sample replicates (columns) harvested at various timepoints following PZQ treatment of infected mice, as indicated by the color bar at top and the scale at right. These results were obtained by re-analysis of the RNA-Seq data from McCusker et al. [1] using the *S. mansoni* lncRNA transcriptome published by Maciel et al. [2] as reference. Gene expression levels were measured by RNA-seq and are shown as Z-scores, which are the number of standard deviations below (blue, downregulated) or above (red, upregulated) the mean expression value among treated and control samples for each gene, as indicated by the scale at right. A total of 1,431 Smps showed statistically significant differential expression (FDR < 0.05).

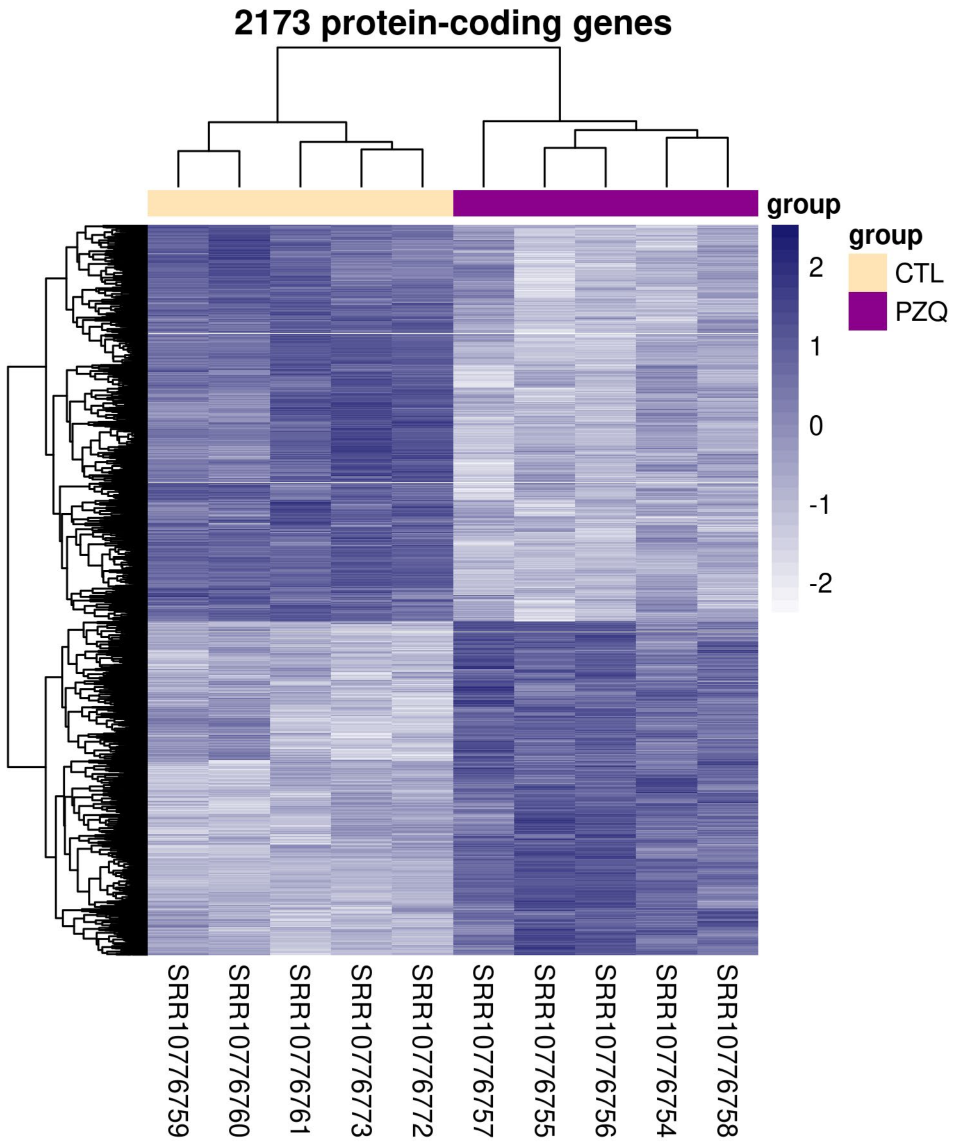

Supplementary Figure S3. Heatmaps of differentially expressed protein-coding genes (Smps) detected by RNA-seq of adult worm couples obtained from mice 14 h after treatment with a single, sub-lethal dose of PZQ (100 mg/kg) delivered by intraperitoneal injection at 4 weeks post-infection. Hierarchical clustering of differentially expressed Smps (lines) in 5 adult worm sample replicates (columns) harvested 14 h following praziquantel (PZQ) treatment of infected mice or in 5 replicates of control (CTL) worms, as indicated by the color bars at the top and the color legends at right. These results were obtained by re-analysis of the RNA-Seq data from McCusker et al. [1] using the *S. mansoni* lncRNA transcriptome published by Maciel et al. [2] as reference. Gene expression levels were measured by RNA-seq and are shown as Z-scores, which are the number of standard deviations below (downregulated, light blue) or above (upregulated, dark blue) the mean expression value among treated (PZQ) and control (CTL) samples for each gene. A total of 2,173 Smps were considered significantly differentially expressed (FDR < 0.05).

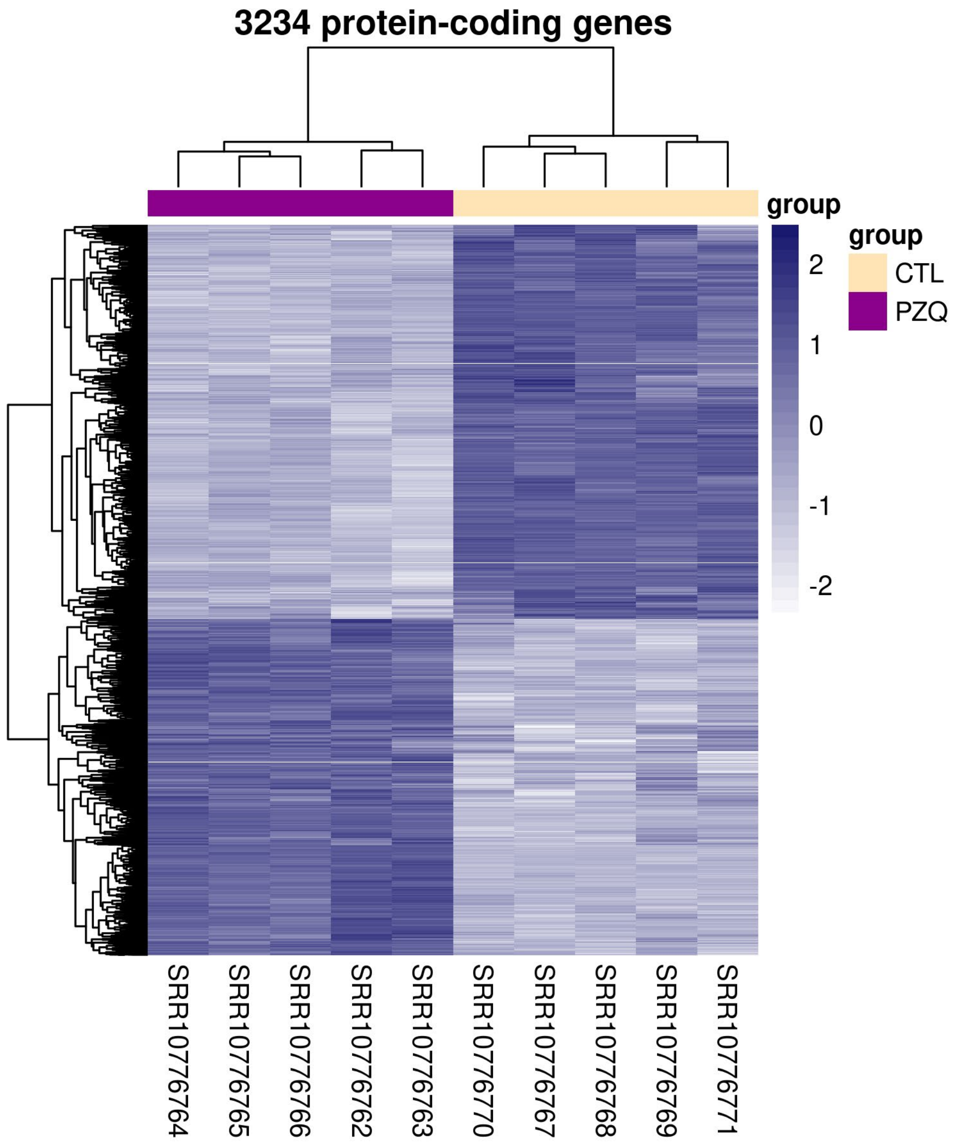

Supplementary Figure S4. Heatmaps of differentially expressed protein-coding genes (Smps) detected by RNA-seq of adult worm couples obtained from mice 14 h after treatment with a single, sub-lethal dose of PZQ (100 mg/kg) delivered by intraperitoneal injection at 7 weeks post-infection. Hierarchical clustering of differentially expressed Smps (lines) in 5 adult worm sample replicates (columns) harvested 14 h following praziquantel (PZQ) treatment of infected mice, or in 5 replicates of control (CTL) worms, as indicated by the color bars at the top and the color legends at right. These results were obtained by re-analysis of the RNA-Seq data from McCusker et al. [1] using the *S. mansoni* lncRNA transcriptome published by Maciel et al. [2] as reference. Gene expression levels were measured by RNA-seq and are shown as Z-scores, which are the number of standard deviations below (downregulated, light blue) or above (upregulated, dark blue) the mean expression value among treated (PZQ) and control (CTL) samples for each gene. A total of 3,234 Smps were considered significantly differentially expressed (FDR < 0.05).

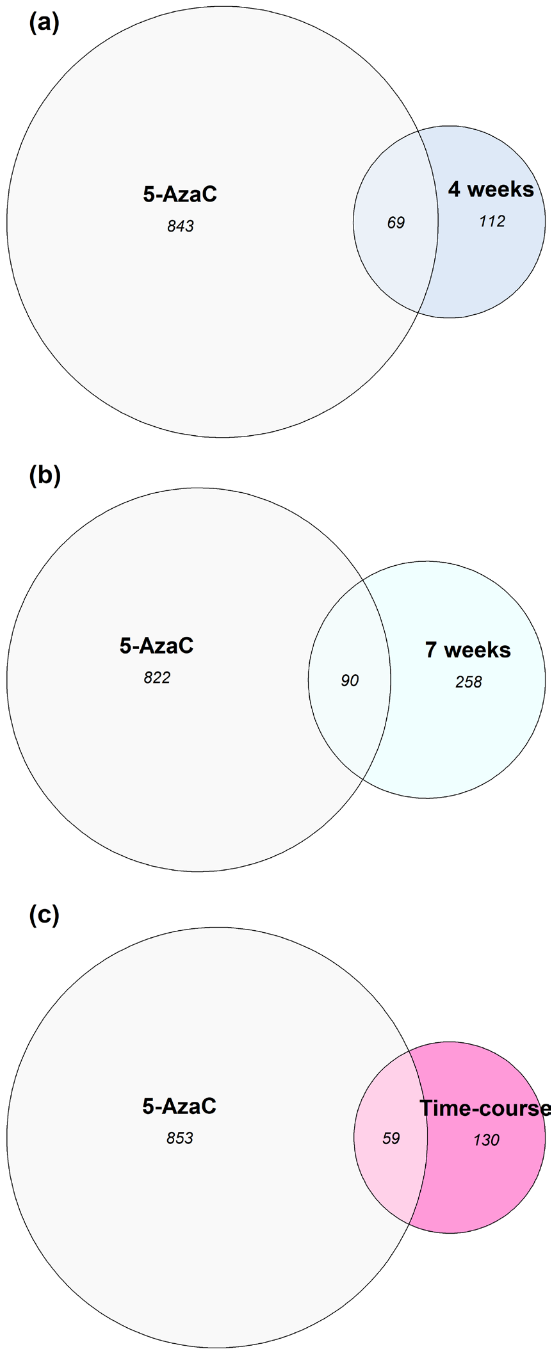

Supplementary Figure S5. Analysis of long non-coding RNAs (lncRNAs) differentially expressed upon distinct praziquantel (PZQ) treatment regimens and 5-azacytidine (5-AzaC) treatment. The Venn diagrams show the number of lncRNAs that were detected as differentially expressed in one experiment alone, or at the same time with "5-AzaC" experiment (adult worm pairs treated *in vitro* with 5-AzaC at 491 µM for 48 h [3]). (**a**) RNA-Seq from adult worm couples obtained from mice 14 h after treatment with a single, sub-lethal dose of PZQ (100 mg/kg) delivered by intraperitoneal injection at 4 weeks post-infection ("4 weeks" experiment); (**b**) RNA-Seq from adult worm couples obtained from mice 14 h after treatment with a single, sub-lethal dose of PZQ (100 mg/kg) delivered by intraperitoneal injection at 7 weeks post-infection ("7 weeks" experiment); and (**c**) RNA-Seq from adult worm couples obtained from mice across 10 time points at 0, 0.25, 1, 3, 6, 9, 12, 24, 48 and 96 h after treatment with a single, curative dose of PZQ (400 mg/kg) delivered by oral gavage at 7 weeks post-infection ("Time-course" experiment).

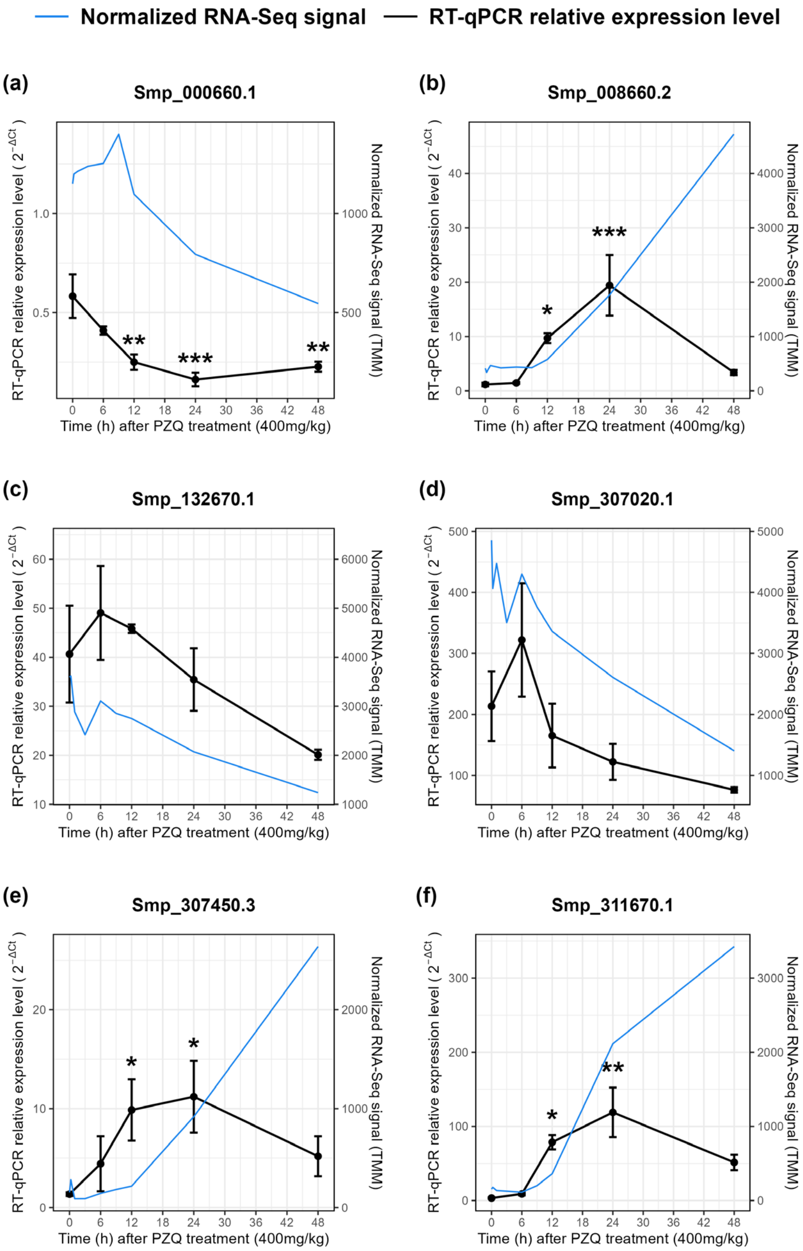

Supplementary Figure S6. Validation by RT-qPCR of protein-coding genes (Smps) detected as differentially expressed upon praziquantel (PZQ) treatment *in vivo*. Six Smps detected by RNA-Seq as differentially expressed simultaneously in all three PZQ treatment regimens were selected for *in vitro* RT-qPCR assays, namely (**a**) Smp_000660.1, (**b**) Smp_008660.2, (**c**) Smp_132670.1, (**d**) Smp_307020.1, (**e**) Smp_307450.3, and (**f**) Smp_311670.1. For each of the six selected Smps, the expression profiles obtained with RNA-Seq are shown on the right (blue line) as TMM (trimmed mean of M values), whereas the RT-qPCR results are shown on the left (black line). For the RT-qPCR data, mean ± SEM from six biological replicates are shown, and a linear mixed-effects statistical model was applied. *$p < 0.05$, **$p < 0.01$, ***$p < 0.001$.

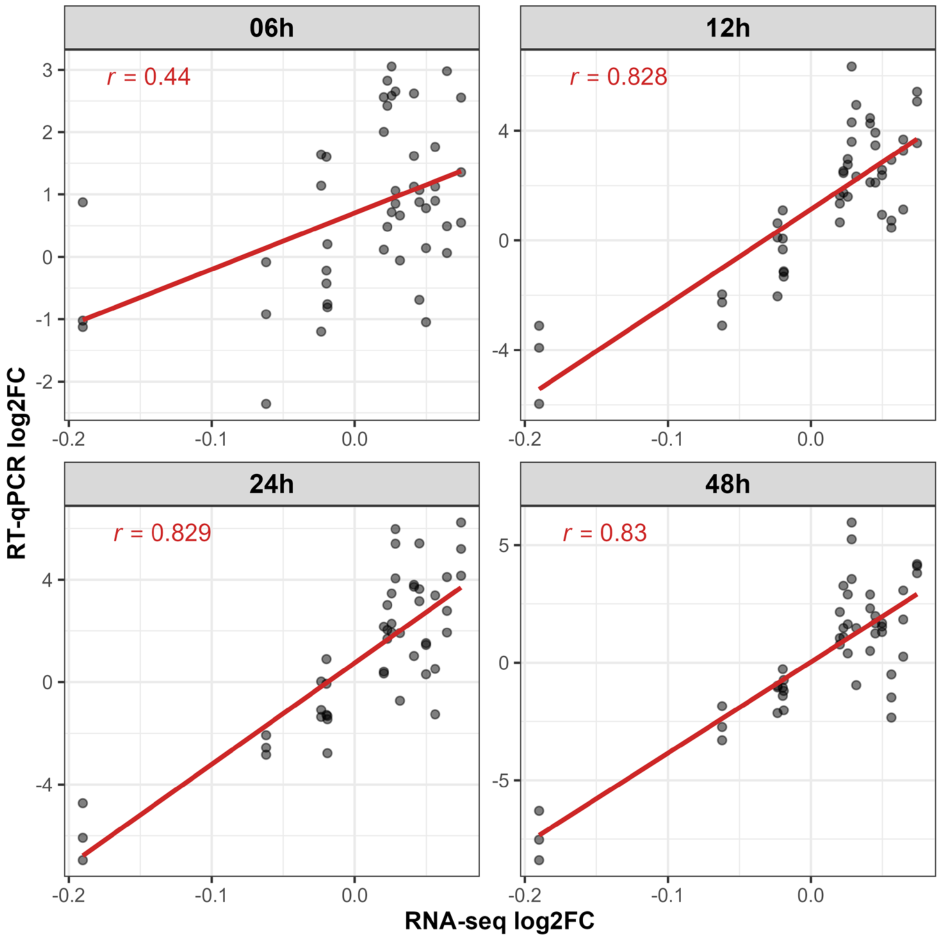

Supplementary Figure S7. Correlation between RNA-Seq and RT-qPCR analyses. Pearson correlation between the fold changes (FC, grey dots) in expression measured by RNA-seq or RT-qPCR of sixteen selected genes (ten lincRNAs and six protein-coding genes); fold changes were obtained by measuring the expression level of each gene in adult worm couples obtained from mice across 5 timepoints at 0, 6, 12, 24, and 48 h after treatment with a single, curative dose of PZQ (400 mg/kg) delivered by oral gavage at 7 weeks post-infection. Log2FC of the sixteen genes obtained with the RNA-Seq assay is represented in the x-axis, and log2FC of the sixteen genes obtained with RT-qPCR is represented in the y-axis. r: Pearson correlation coefficient.

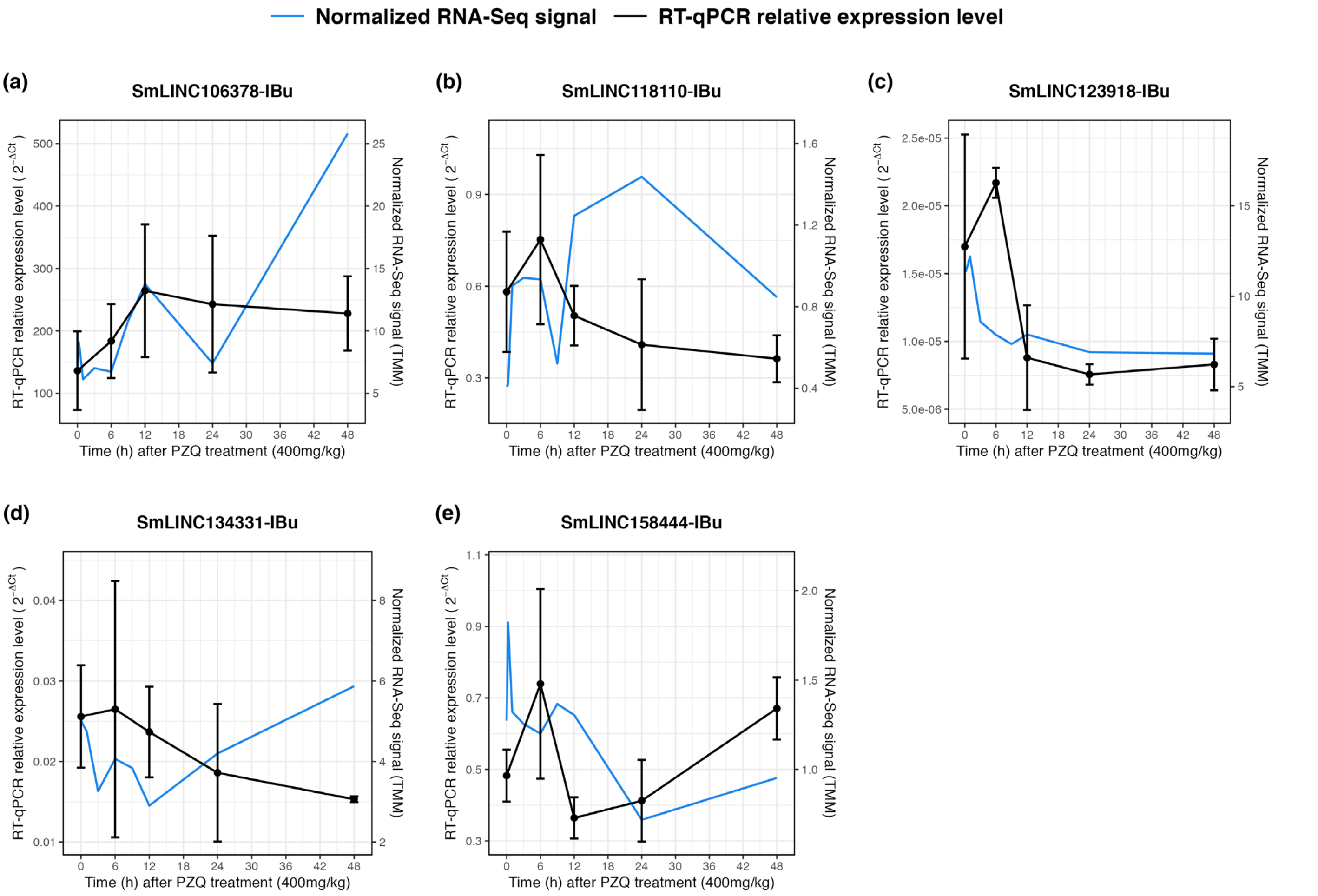

Supplementary Figure S8. RT-qPCR results of long non-coding RNAs (lncRNAs) detected as non-differentially expressed upon praziquantel (PZQ) treatment *in vivo*. Five lncRNAs detected by RNA-Seq as non-differentially expressed simultaneously in all three PZQ treatment regimens were selected for *in vitro* RT-qPCR assays as a secondary control for validation of differentially expressed lncRNAs, namely (**a**) SmLINC106378-IBu, (**b**) SmLINC118110-IBu, (**c**) SmLINC123918-IBu, (**d**) SmLINC134331-IBu, and (**e**) SmLINC158444-IBu. For each of the five tested lncRNAs, the expression profiles obtained with RNA-Seq are shown on the right (blue line) as TMM (trimmed mean of M values), whereas the RT-qPCR results are shown on the left (black line). For the RT-qPCR data, mean ± SEM from six biological replicates are shown, and a linear mixed-effects statistical model was applied. None of the timepoints were statistically significant, confirming that the lncRNAs are not differentially expressed.

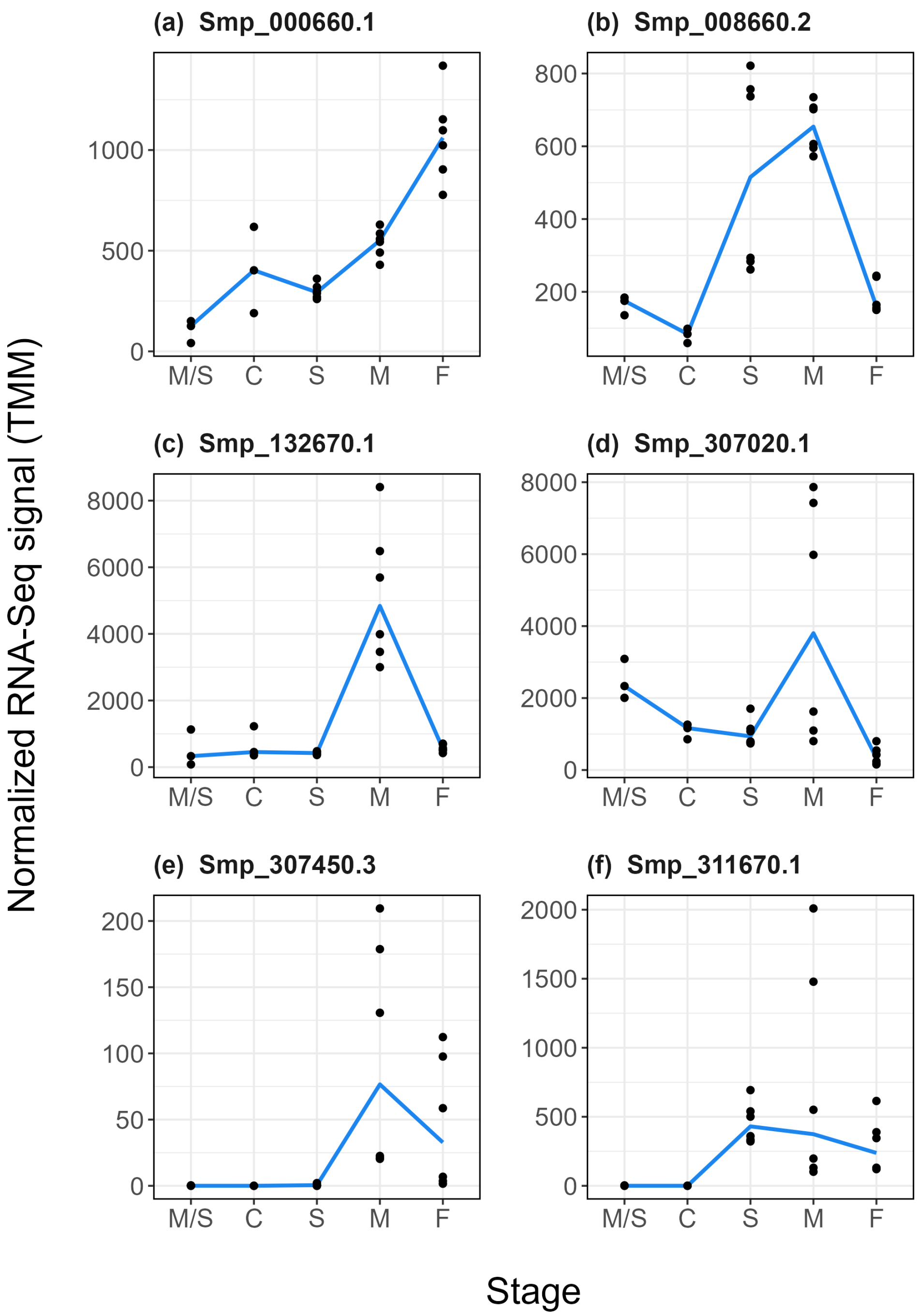

Supplementary Figure S9. RNA-seq expression profiles of selected protein-coding genes (Smps) measured in control assays at different *S. mansoni* life-cycle stages. The expression levels of six selected Smps are shown, (**a**) Smp_000660.1, (**b**) Smp_008660.2, (**c**) Smp_132670.1, (**d**) Smp_307020.1, (**e**) Smp_307450.3, and (**f**) Smp_311670.1. These *S. mansoni* Smps were selected after re-analysis of RNA-Seq public datasets of parasites collected from mice treated with PZQ in the "time-course experiment" of McCusker et al. [1]. The y-axis shows the expression level (shown as TMM, trimmed mean of M values) for each Smp in control RNA-seq assays, as compiled by Silveira et al. [4], at the stage indicated in the x-axis as follows: miracidia/sporocysts (M/S), cercariae (C), schistosomula (S), adult males (M), and adult females (F).

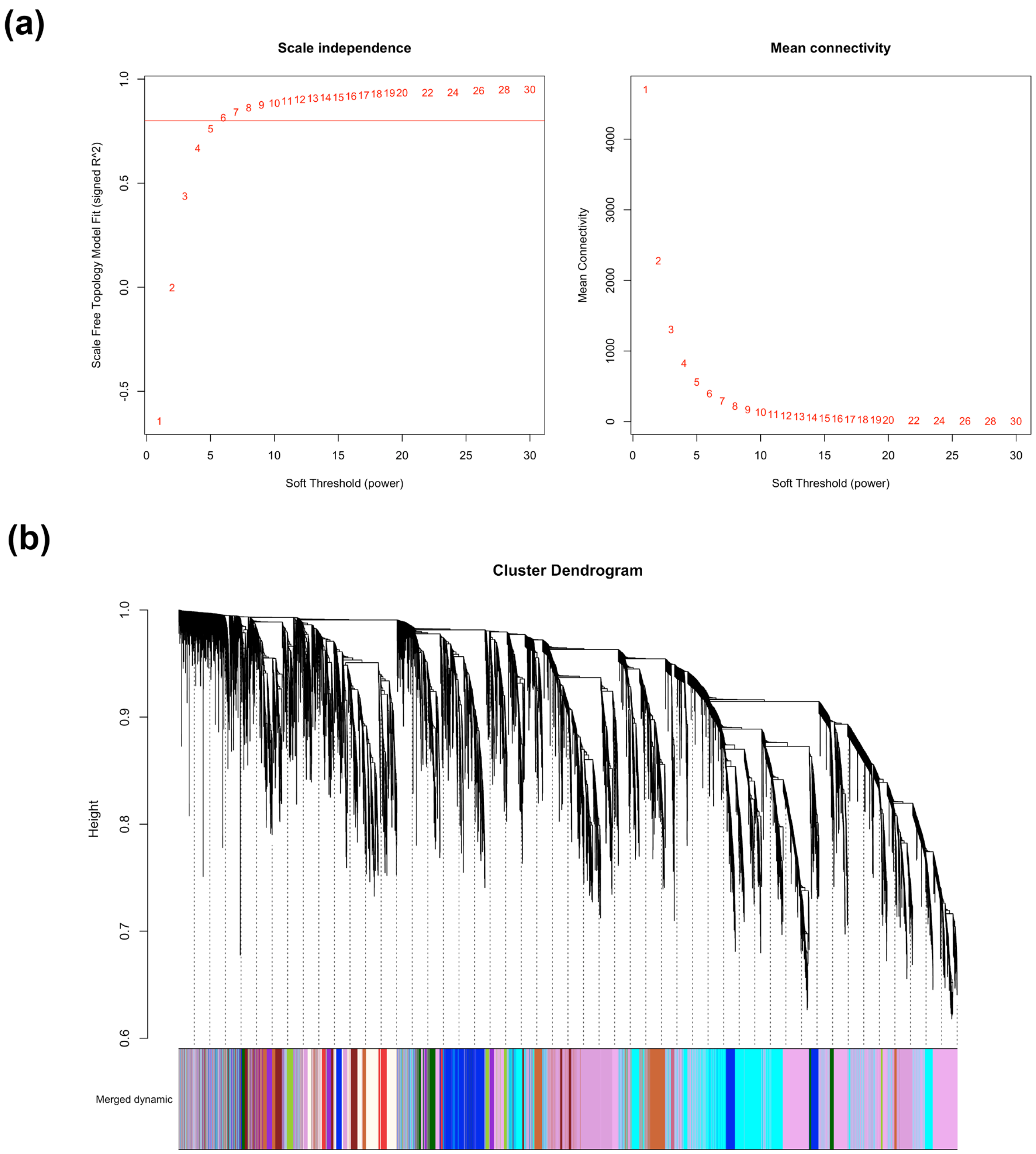

Supplementary Figure S10. Weighted Gene Co-Expression Network Analysis (WGCNA) parameters and results for the RNA-Seq data. Using the RNA-Seq data for the "Time-course", "4-weeks", and "7-weeks" experiments a WGCNA was performed to identify co-expression modules. (**a**) Based on the Scale-free topology model fit index (Scale independence; y-axis, left plot) and Mean connectivity (y-axis, right plot) in function of soft-threshold powers (x-axis), shown as numbers inside each plot, a soft-threshold power equal to 9 was selected for module construction. The red line marks adjusted $R^2 \geq 0.8$. (**b**) Hierarchical cluster dendrogram shows the 15 co-expression modules (assigned with the colors at the bottom) identified based on a dissimilarity measure of the Topological Overlap Matrix (1 – TOM), and merged based on modules' eigengenes dissimilarity, with a 0.40 threshold.

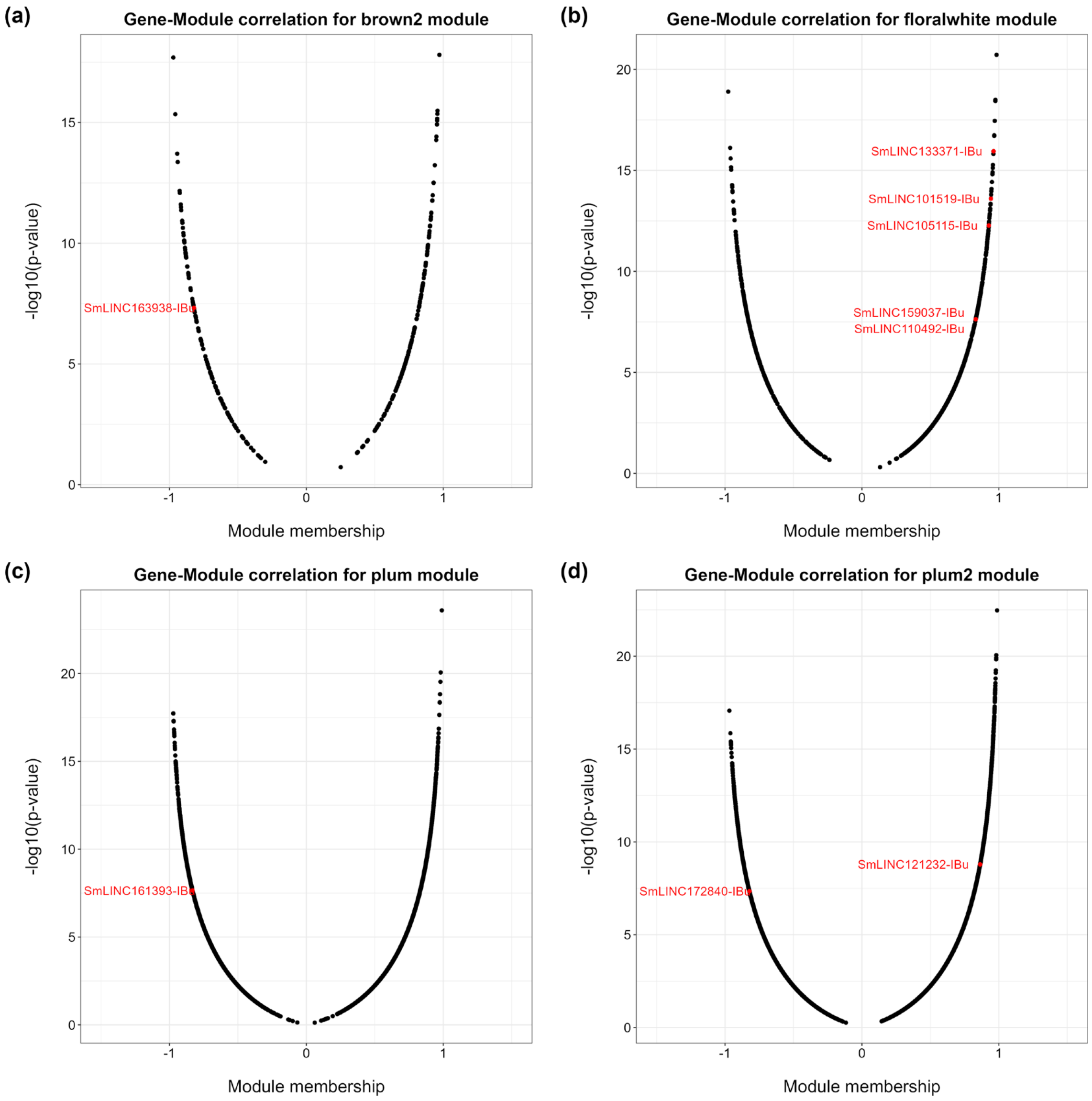

Supplementary Figure S11. Gene-Module correlation for the four modules containing the differentially expressed long non-coding RNAs (lncRNAs) tested by RT-qPCR. The plots show the Pearson's correlation between gene expression data from RNA-Seq and their corresponding module eigengene (Module membership, x-axis), and the $-\log_{10}$ p-values (y-axis) calculated for these associations. The four modules displayed are: (**a**) "brown2", (**b**) "floralwhite", (**c**) "plum", and (**d**) "plum2". The RT-qPCR-tested lncRNAs are highlighted in red.

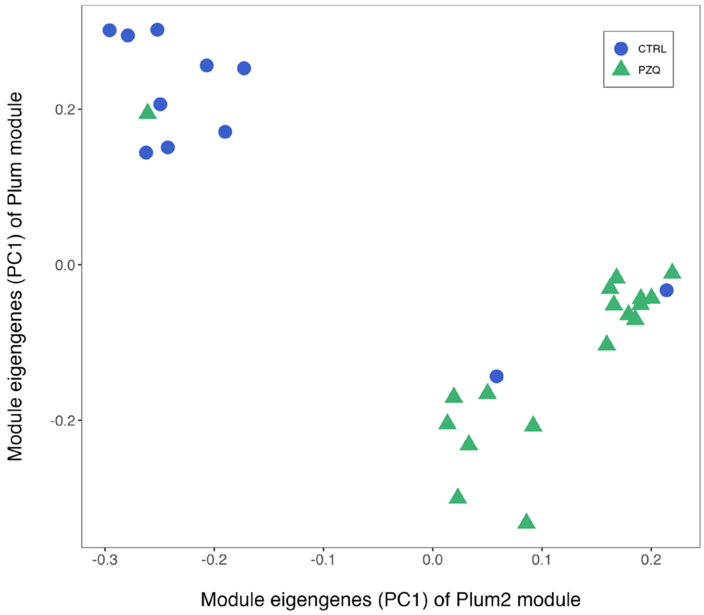

Supplementary Figure S12. Clustering of RNA-Seq biological replicates assessed by modules eigengenes values. The RNA-Seq samples were analyzed with WGCNA, resulting in 15 co-expression modules. Two of those modules were identified with the highest association with praziquantel treatment, relative to control groups: "plum" (R^2 = 0.53, p-value = 7.34e-06), and "plum2" (and R^2 = 0.52, p-value = 1.13e-05). The plot displays the eigengene values (i.e. the first principal component, PC1, of the expression matrix of the corresponding module) for these two modules for each of the RNA-Seq samples, which separates them into two distinct clusters: praziquantel treated (PZQ; green triangles), and control group (CTRL; blue circles).

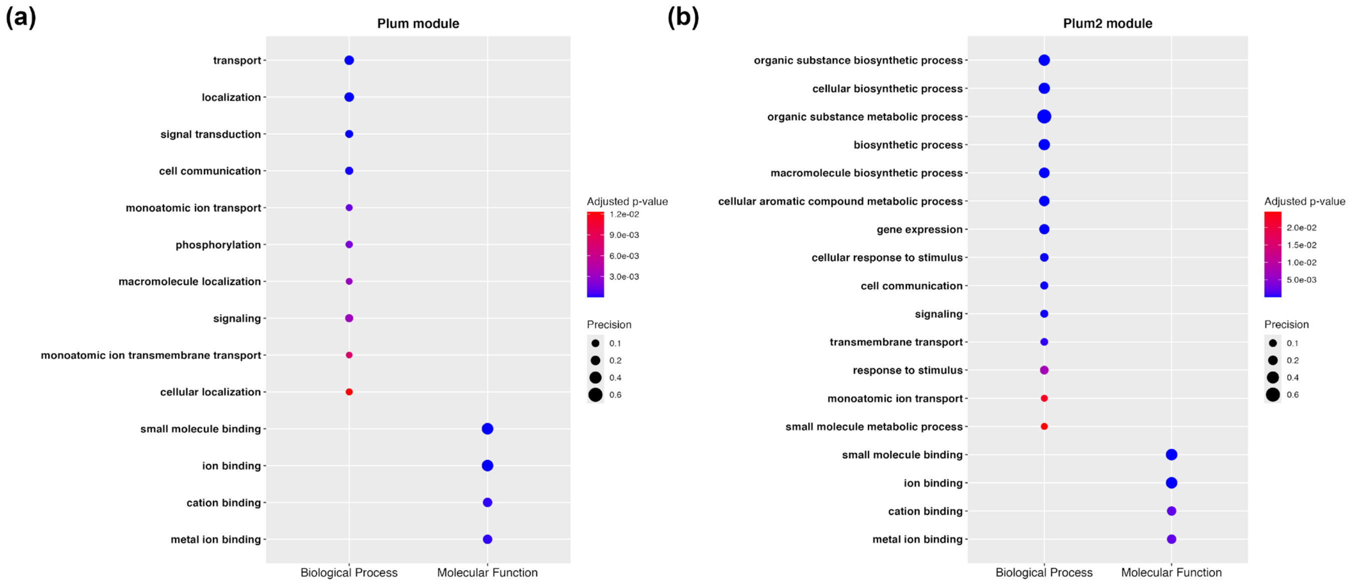

Supplementary Figure S13. Functional profiling of the protein-coding genes inside “plum” and “plum2” modules. Functional profiling analysis was performed with gProfiler2 v0.2.3 [5] with g:SCS multiple testing correction method, and redundancy was removed using REVIGO v1.8.1 [6]. The most significantly enriched and relevant Gene Ontology (GO) terms, including Biological Process and Molecular Function terms, are shown for the (**a**) “plum”, and (**b**) “plum2” modules. The size of each point is proportional to the precision, also known as gene ratio (i.e. proportion of genes in the input list that are annotated to the function). The colors show the statistical significance of the enrichment.

References

1. McCusker, P., C.M. Rohr, and J.D. Chan, *Schistosoma mansoni alter transcription of immunomodulatory gene products following in vivo praziquantel exposure.* PLoS Negl Trop Dis, 2021. **15**(3): p. e0009200.
2. Maciel, L.F., et al., *Weighted Gene Co-Expression Analyses Point to Long Non-Coding RNA Hub Genes at Different Schistosoma mansoni Life-Cycle Stages.* Front Genet, 2019. **10**: p. 823.
3. Amaral, M.S., et al., *Long non-coding RNA levels can be modulated by 5-azacytidine in Schistosoma mansoni.* Sci Rep, 2020. **10**(1): p. 21565.
4. Silveira, G.O., et al., *Assessment of reference genes at six different developmental stages of Schistosoma mansoni for quantitative RT-PCR.* Sci Rep, 2021. **11**(1): p. 16816.
5. Kolberg, L., et al., *gprofiler2 -- an R package for gene list functional enrichment analysis and namespace conversion toolset g:Profiler.* F1000Res, 2020. **9**.
6. Supek, F., et al., *REVIGO summarizes and visualizes long lists of gene ontology terms.* PLoS One, 2011. **6**(7): p. e21800.
